# Litigation and political transformation: the case of Russia

**DOI:** 10.1007/s11186-018-9327-5

**Published:** 2018-09-27

**Authors:** Chris Thornhill, Maria Smirnova

**Affiliations:** School of Law, Williamson Building, University of Manchester, Oxford Road, Manchester, M13 9PL UK

**Keywords:** Administrative law, Hybrid polities, Institution building, Litigation, Russian Federation, Social integration

## Abstract

This article analyzes some recent developments in the system of public law in the Russian Federation, focusing in particular on changing patterns of litigation and increases in use of administrative law, linked to new acts of legislation. It argues that discussion of the Russian case provides a sociological perspective in which we can understand the importance of legal actions in hybrid polities. It explains that litigation in Russia, even where it may have counter-systemic outcomes, is partly incentivized by the government, as promotion of access to law is seen as a means to formalize interactions between citizens and government and so to extend the societal penetration of the political system more generally. Litigation thus forms a mode of practice that, dialectically, possesses both inner- and counter-systemic status. In addition, the article argues that the case of Russia allows us to comprehend litigation as an element in processes of nation building and social integration more widely, and Russia illuminates the systemic significance of litigation in other societies.

It is widely observed that litigation can lead to political change. Indeed, there now exists a large body of legal-sociological research that is engaged with the correlation between litigation and political transformation. Above all, such research examines ways in which, in different societies, changing patterns of litigation, especially litigation concerning human rights law, can establish new laws and new policies, and even shape the basic architecture of national constitutions. Broadly speaking, this research can be divided into the following lines of argument.

First, some leading perspectives in research on litigation focus on conventional processes of *legal mobilization*, and, most particularly, they analyze strategic litigation as an institutionally transformative practice, shaping public policy and governance structures.^1^ Earlier research in this area was mainly focused on progressive legal politics in the United States, especially in the civil rights era.^2^ This research addressed ways in which select social groups pursued litigation to develop and expand distinctive rights, using litigation over single rights to alter the broad terms of articulation between groups of citizens and the government.^3^ This approach has been extended to examine litigation for rights as a distinct pattern of *collective citizenship*, supplementing more typical modes of representation, giving rise to clearly political legislative outcomes.^4^

Second, important analyses of rights-based litigation have examined litigation in societies in which opportunities for more direct contention are constrained, usually for political reasons. In such settings, these analyses have observed that some patterns of litigation form a channel for expressing contentious culture, at times creating openings for radical political re-orientation (Sikkink 1993; Moustafa 2003; Smulovitz 2005; El-Ghobashy 2008; Bernard-Maugiron 2008). On such accounts, litigation forms a parallel mode of political agency, which compensates for the lack of opportunities in the (strictly defined) political arena (Hualing and Cullen 2001; O’Brien and Li 2004).

Third, some of most significant research on the politics of litigation engages with changing patterns of litigation from a perspective based in analysis of legal consciousness, arguing that rising litigation concerning human rights results from the spread of legal awareness through society (Merry 1990). Importantly, leading proponents of this approach attach great importance to the role of international law in litigation, explaining that contested or rights-related litigation is often triggered by reception of international human rights law, articulating transnational patterns of citizenship (Risse and Sikkink 1999; Lutz and Sikkink 2000; Merry 2005).

Fourth, salient perspectives in this research have addressed the role of rights-related litigation in societies that have recently undergone transition to democracy, either from civil conflict or political authoritarianism. Such perspectives view human rights litigation as a practice that actively transforms and solidifies democratic institutions, stressing that the willingness of citizens to use the law to claim rights reflects growing confidence in public authorities, and demonstrates the rising stability of democracy (Giles and Lancaster 1989; Garavito and Franco 2000; Latorre Iglesias 2015; Ripoll and Sandvik 2015).

Naturally, it is not possible to approach this complex body of research as a fully homogenous set of outlooks, and the variations between these approaches are quite clear. However, these different lines of analysis are connected by a number of shared preconditions. Notably, these analyses are linked by the fact that they interpret litigation for rights as a clearly oppositional activity.^5^ To be sure, some of the literature on the United States, in particular, has explained that litigation can easily acquire a dialectical dimension and that it can act to underpin the extension of state structures across society.^6^ Across the spectrum, however, perspectives in this research claim that rising quantities of rights-related litigation reflect positional struggles on the part of distinct social groups, originating in anti-systemic attitudes or strategies. Indeed, each sub-field outlined above is founded in essentially conflict-sociological approaches to litigation, based either in sociological theories of political mobilization or sociological theories of contentious collective action.^7^

This article adds to these lines of legal-sociological research on the politics of litigation by analyzing the political implications of rights-related litigation in Russia. In the first instance, Russia may not appear a promising focus for such inquiry. Use of courts in Russia is often treated sceptically in academic commentaries, and there remains a tendency to dismiss judicial procedures in Russia as subject to political manipulation.^8^ This means that litigation in Russia is not commonly observed as a politically formative process.^9^ In fact, however, Russia provides a singularly illuminating context for addressing the sociological and political implications of litigation, and it allows us to expand this field in several ways. Owing to the distinctive political environment in which litigation occurs, the Russian context challenges standard sociological perspectives on litigation, and it demands a new explanatory frame for addressing the politics of litigation.

Thee are several key reasons why Russia needs to be included on research on litigation.

First, Russia possesses many characteristics that are identified in the wider body of research on litigation, and, in recent years, Russian society has seen rapidly rising public use of courts, for different types of litigation (i.e., both civil and administrative).^10^ In particular, recent years have witnessed a very significant increase in administrative litigation, and—notably—in proceedings filed against public agencies for human rights violations.^11^ For example, suits filed because of illegal actions (and illegal inactions) of state and municipal bodies increased by circa 300% between 2006 and 2011.^12^ Moreover, increasing litigation has been flanked by a growth in the use of international law in domestic proceedings, especially in administrative law cases. In consequence, litigation has also acquired far-reaching constitutional results. By any reasonable indicator, therefore, Russia is a society in which increased litigation is a prominent socio-political phenomenon, albeit, as discussed below, in distinctive form.

Second, litigation in Russia has assumed a significance that is rather different from its role in other polities. Importantly, Russia falls between the categories of polity usually examined in research on litigation. Increasingly, it is common to see Russia as a simply authoritarian polity (Hassner 2008; Petrone 2011; Chandler 2014; Gill 2015; Gelman 2015; Hale 2015), or at least as an embodiment of some variant on electoral authoritarianism.^13^ To be sure, it is not difficult to find features in Russian government that justify this classification; these include, in particular, controlled access to resources of political competition, weak institutionalization of opportunities for party-political opposition, use of patronage to generate regional support for governmental policies. In some respects, however, Russia does not conform to a fully authoritarian model, although its authoritarian features remain undeniable. In particular, for a perspective that examines legal politics, the category of the *hybrid democratic-authoritarian state* still retains explanatory value in Russia, and Russia can still be positioned in a ‘messy middle ground’ between regime types.^14^ Indicators employed to calibrate the position of the legal system in partially democratized polities imply that Russia cannot be ranked without qualification at the authoritarian end of the spectrum of regime types.^15^ If we place emphasis on the political role of legal practices in Russia, we can see that the Russian polity contains intricately connected, and intrinsically hybrid, elements, which are simultaneously authoritarian and progressive.^16^ In the field of legal politics, ostensibly authoritarian policies in Russia can at times have very unintended outcmes, acting as sources of alternative norm construction within the governance system. Under some circumstances, in fact, such policies lead to the build-up of counter-weights to governmental authority. Discussion of litigation thus indicates that a new categorization is required for the Russian polity, and analysis of litigation in Russia, especially human rights litigation, reveals unusual forms of hybridity in the political system. As mentioned, analytical perspectives have been estabished for observing the role of human rights litigation in democratic and transitional polities. As yet, however, we lack a broad model for addressing such litigation in hybrid polities, and a distinctive paradigm is required to capture this. Discussion of Russia allows us to construct the complex causes and outcomes of litigation in hybrid polities.

Third, analysis of the politics of litigation in Russia, as a hybrid state, brings into relief certain less visible characteristics of rights-related litigation more generally. In fact, discussion of changing patterns of litigation in the Russian context allows us to identify certain more common features of litigation, which are less immediately manifest in polities with more solidly democratic institutions. Notably, analysis of Russia adds weight to the perception, which is at best marginally implied in other research, that increases in litigation can reflect clearly systemic functions^17^: namely, that, under some conditions, government actors will intentionally stimulate litigation because this helps to expand state capacity, and it creates a legal framework that consolidates governance functions across national society. In consequence, analysis of Russia provides deep corroboration of the insights contained in research that imputes systemically formative importance to litigation. Analysis of Russia intensifies the validity of such research because it explains that the systemic benefits of litigation can be so great that even hybrid governments, usually inclined to restrict independent political challenges, will recognize these benefits, and they will accept criticism transmitted through legal actions as part of a compensatory calculation. Interpretation of patterns of litigation in Russia requires relativization of the common assumption that increasing litigation is explicable as a counter-systemic, oppositional social practice. In Russia, rising litigation, especially for basic rights, can be observed as a process, which, in part, is originally triggered by systemic imperatives, and increases in litigation are clearly determined by governmental policies. Once initiated, however, litigation can also acquire a path-dependent, potentially counter-systemic autonomy. In this respect, unexpectedly, analysis of Russia qua hybrid polity produces insights that allow us to understand the political implications of litigation more generally, across different societies and different polity types.

On this basis, this article is designed to outline an approach for examining the causes and functions of litigation in Russia, as a sui-generis, hybrid state, and it interprets recent changes in litigation practices within a broad institutional context. However, this article also argues that analysis of litigation in Russia challenges us to revise the sociological paradigms that are commonly used for evaluating litigation and its consequences. On one hand, it argues that, to explain litigation in this context, we should not necessarily view litigation as a counter-systemic practice, and we need to emphasize, not only contentious behaviours, but also *systemic functions*, as factors that influence the growth of litigation, especially in public law. On the other hand, it argues that, even when triggered by official policies, litigation always contains a transformative dimemsion, and government bodies are forced to accept transformative outcomes in order to acquire the benefits that they identify in litigation. The article concludes by suggesting ways in which this paradigm might help to explain the politics of litigation more generally.

## Policy background

Recent decades in Russia have seen many attempts to improve the performance of judicial institutions, and to encourage individual agents to resolve individual disputes within the formal legal sphere—in law courts. Such policies have almost invariably been driven by the concern that lack of public confidence in the law has limited state penetration and weakened the effective capacities of government.

Legal reform has a long history in Russia, and improvements to the judicial system have often been seen as means to remedy weaknesses in the political system. It has been repeatedly diagnosed, at different junctures in Russian state formation, that centralized institutions do not possess deep integrational force in society, and that informal linkages, often connected to patrimonial distribution of goods, form a core component of state authority.^18^ Reform to the legal system has often been proposed as a means to correct this institutional informality. In recent history, however, legal reform policies acquired distinct prominence in the last years of the Soviet Union, under Gorbachev, who sought to strengthen the rule of law, to increase use of courts, and, by these means, to improve the quality of government functions (White 1990, p. 37; Kahn 2002, p. 87). After Gorbachev, Yeltsin introduced the Concept of Judicial Reform in 1991, which emphasized the need “to move the courts closer to the people, to facilitate access to justice,” and generally to increase public understanding of the legal system. In 1993, then, Yeltsin passed a decree on access to legal information, which provided for the creation of television programs and “live courtroom” shows, designed to educate people in litigation.^19^ By 1999, a nation-wide program was established to ensure access to legal information in public libraries. Today, this network operates in 1424 public libraries in 77 Russian regions.^20^ These centers have dramatically increased the access of Russian citizens, even in the most remote regions, to judicial institutions, and to general legal knowledge.

After 2000, such policies were extended by Putin, who introduced a large package of judicial reforms, which were also intended, in part, to facilitate access of citizens to courts and to promote public confidence in legal institutions. Central to these reforms was legislation designed to clarify procedural rules for court hearings,^21^ and to minimize litigation costs,^22^ which clearly increased legal opportunities for Russian citizens.^23^ Federal target programs were also created to increase transparency and accessibility of courts.^24^ These programs guaranteed public access to courtrooms, and, to ensure judicial openness, they established free access of journalists to courts as a justiciable right. In 2009, a special federal law was passed to ensure open access to information about the functions of the judicial system.^25^ Significantly, in a related ruling, the Supreme Court stressed that open access to judicial information enhances “public control over the functioning of the judiciary,” and it serves to maintain public confidence in courts.^26^ As a result of these policies, between 2008 and 2011, there was a 4000 percentage increase in the number of cases in all federal courts of general jurisdiction,^27^ constituting 90% of all courts in Russia, that were made available for public access via open databases.

Of particular relevance in these processes is the fact that these judicial reforms have included distinct measures to facilitate administrative litigation, especially litigation entailing appeals against acts, decisions, and failings of public bodies that affect individual human rights. In 2002, Putin declared that reinforcement of procedures for human rights protection and access to justice had to be given priority in the judicial system.^28^ He emphasized later that, as a part of a wider policy of administrative reform, it was essential to “create an efficient mechanism for the resolution of disputes between the citizens and the state through enhancement of both administrative procedures and judicial mechanisms.”^29^ Accordingly, 2001–2002 saw the adoption of new procedural codes, most notably the Civil Procedure Code (CPC), but also a code regarding litigation involving business interests (*arbitrazh*). Both these codes served to facilitate administrative litigation, and both contained a chapter systematically regulating the procedures for judicial review. Later, a new Code of Administrative Court Proceedings (CACP), in essence an administrative litigation code, was adopted in 2015,^30^ which makes it easier for citizens to gain access to courts in their disputes with state authorities.^31^ Moreover, policies concerning administrative litigation have established new opportunities for initiating public interest litigation (PIL). In Russia, rules concerning PIL remain restrictive, and they still reflect traces of Soviet-era political paternalism. Generally, the traditional German model for PIL is adopted, which prevents “private parties from litigating on behalf of collective public interests” (Greve 1989, p. 197). Recent legislation, however, has widened legal opportunities for PIL, and it allows a number of proxies to file suit against public bodies. In 2014, most importantly, a new Federal Law “On Citizens’ Oversight” was adopted, which authorizes different associations, including NGOs, “to submit claims to court against public bodies in the interests of an unidentifiable number of persons.”^32^

Significantly, judicial and procedural reforms in Russia have been closely linked to the incorporation of international law, and especially international human rights law, in Russian domestic law. Generally, Article 15(4) of the Russian Constitution and relevant Supreme Court Plenary Rulings dictate that international law must be directly applied in court practice.^33^ More specifically, however, all the new procedural codes instruct the courts to resolve disputes by referring to international treaties, alongside relevant domestic legislation.^34^ Moreover, both the regular courts and the Supreme Court now systematically take into account relevant practice of the European Court of Human Rights (ECtHR).^35^ The Supreme Court regularly refers to the ECHR in order to establish normative uniformity in Russian courts.^36^ This is especially important in cases concerning access to justice. Pilot judgments of the ECtHR concerning access to courts are implemented on a national scale.^37^ International obligations concerning access to courts have led to important procedural developments in the Russian judicial system. For example, some of the legislation promoting transparency of courts and litigation is a result of international cooperation with the Council of Europe.^38^ The push for improved access to law, therefore, is partly framed by international law.

This analysis demonstrates that access to law is a priority policy objective of public agencies in Russia. In fact, government policies are clearly oriented toward *demand-stimulation* for law, and they are designed to encourage social agents to conduct disputes in court. Further, policies concerned with increasing access to law have attached particular weight to the domain of public law, and they are intended to ensure that disputes between citizens and government agents are regulated in formal procedures, often using human rights law. This in turn leads to a strong interaction between domestic and international law, such that international law assumes a constitutionally reinforced position in the domestic polity.

## Rising volume of litigation

### General litigation

As mentioned, these policies have been accompanied by a significant increase in litigation in Russia. The general volume of civil and administrative litigation in Russia began to rise very noticeably after 2001–2002, as a result of judicial reform programmes and the adoption of the new procedural codes. In 2005, the Russian Congress of Judges reported that courts had that year processed what was then an unusually high annual sum of 5,000,000 civil cases.^39^ This number doubled in the period 2005–2008. By 2016, the courts of general jurisdiction in Russia heard more than 17,000,000 civil and administrative cases per year, almost twice as many as in 2008. Legislative changes also resulted in a rapid growth of claims in *arbitrazh* courts.^40^ Non-normative acts were challenged in *arbitrazh* courts at a similarly increasing rate: a tenfold increase occurred between 2000 and 2013. Also, from 2007 on, the number of individual civil and administrative claims in courts of general jurisdiction against all organizations, including state bodies, increased by 36%, from 1,300,000 to 2,100,000.

### Administrative litigation

As part of this general rise in litigation, judicial reform policies have led to significant increases in administrative litigation in Russia, the consequences of which require particular attention.

In addressing this, first, it needs to be noted that, from a doctrinal point of view, the term “administrative litigation” is only now being defined in Russia as a separate type of legal procedure. Traditionally, the term “administrative litigation,” the nearest analogue to which in Russian is “administrative judicial proceedings” (*administrativnoye sudoproizvodstvo*), was used to describe court proceedings concerning administrative offenses (that is, non-criminal offenses against public interests, such as traffic offenses or violation of rules regulating public gatherings).^41^ However, since adoption of the procedural codes in 2001–2002, administrative litigation has increasingly been defined in terms close to its meaning in other jurisdictions. This concept is now used in doctrinal texts to categorize “claims connected with unjustified exercise of power by a public body or an unjustified refusal to exercise power” (Bakhrakh 2008, p. 120).  As a result, administrative litigation is now established as a quite specific type of litigation.^42^ Further, judicial review and administrative litigation are now increasingly connected (Lebedev 2000). From a procedural point of view, moreover, it is not easy in Russia to separate cases of administrative litigation from other cases. Only since 2015 has litigation concerning public actions been formally recognised, not as a specific type of civil litigation, but, distinctively, as administrative litigation.

Despite these classificatory problems, it is still possible to assess the volume of administrative litigation in Russia.

Currently, each year approximately 200,000 cases arising from administrative and public law relations are considered by courts of general jurisdiction.^43^ Among these, nearly 2300 cases requiring judicial scrutiny of federal and regional legislation are heard by courts. This number began to increase shortly after the procedural codes were adopted, and it peaked, at 6000, in 2007. Since then, it has been gradually decreasing. Also, from 2001 to 2007, the number of claims considered by *arbitrazh* courts grew rapidly. It peaked in 2009, at 1715 cases per year. On average, 70% of claims are successful in courts of general jurisdiction. In *arbitrazh* courts, only 26% of such cases are successful. It should be noted that since adoption of the CACP in 2015, the *arbitrazh* courts no longer conduct judicial review. In 2015, the last 464 cases were considered.

Alongside this, there has also been an increase in the number of cases in which individuals and organizations have challenged non-normative administrative acts on grounds that these acts violate their basic rights, defined under the Constitution and current legislation, in accordance with international human rights law. This category of administrative claim occurs in a large variety of situations. For example, such claims may concern the granting or withdrawal of a license, the illegal actions of a police inspector, or the inaction of a municipality in granting a place for a child in a municipal nursery. The courts of general jurisdiction consider around 110,000 claims per year challenging illegal actions or omissions of federal and municipal bodies. This number underwent a period of rapid and significant growth from 40,000 in 2006 to 140,000 in 2011. However, since 2011, it has slightly decreased, and it now seems to have stabilized. In the same period, the rate of success in these cases fell from 64 to 47%. This levelling out could, of course, indicate that the operations of the executive branch have improved. On the other hand, this could be caused by measures introduced by the government to cut the workload of judicial institutions. Notably, the government has introduced instruments to facilitate extra-judicial dispute resolution through petitions to the relevant supervisory organ.^44^ Additionally, the government has, amongst other measures,^45^ implemented procedures designed to filter out frivolous claims,^46^ to promote incentives for private arbitration,^47^ to facilitate conciliation,^48^ and mediation,^49^ and to establish specialized tribunals.^50^ Importantly, the creation of mechanisms for compulsory pre-judicial conflict resolution for some categories of case has been a priority of state policy since 2006.^51^

## Litigation and constitutional outcomes 1

In these respects, we can see a distinct pattern in the use of the law in Russia. First, we can see that the government deliberately stimulates use of law, and strategies to increase use of law reinforce the standing of human rights norms in the national legal system. Governmental stimulation of use of law then generates increases in litigation, especially in public law and in cases with human rights implications. As discussed below, in some respects, these processes mirror more classical lines of legal mobilization, and acts of litigation serve to create norms that define the public arena, reflecting new lines of articulation between government and society. Second, we can observe that litigation in Russia is linked to the particular features of a hybrid polity, and it often results from a deliberate strategy on the part of government institutions: that is, it is not driven by independent oppositional attitudes, but is induced by governmental prerogatives, which serve particular governmental functions. As a result, litigation rarely takes the form of contentious politics seen in other societies. In fact, the political results of litigation are linked, dialectically, to the semi-authoritarian nature of the governance system.

### Litigation as systemic consolidation

It is not difficult to identify the systemic prerogatives that underlie the increases in litigation in Russia since 2000. Most evidently, litigation is driven by the fact that leading actors within the political system have decided that the lines of interaction between government bodies and agents in different parts of society are too weakly institutionalized. This fact is perceived as undermining the authority of central state institutions and as inhibiting the effective performance of basic governance functions. As a result, government policies are consciously designed to encourage litigation as this is seen to help to impose a stricter legal order on social interactions, and to connect social agents more immediately to government bodies. In this respect, litigation is the direct result of a state-building process, which is intended to harden the linkage between state and society, and to extend the societal penetration of official political institutions. At the core of this strategy is an instrumental view of constitutional law, which presumes that litigation intensifies the impact of constitutional law in society, and it subjects social exchanges, in different domains, to a uniform constitutional grammar, thus expanding state structure into society as a whole. On this basis, the government’s endeavor to stimulate *demand for law* across society is part of a wider strategy to promote access to law as a means to integrate social actors more fully in the political system and to solidify the political system as a formal institutional order.^52^ Litigation, thus, originates as a controlled practice, and, in different ways, it serves to intensify the effective authority of political institutions.^53^

## Systemic consolidation 1: reduction of shadow justice

The significance of litigation as a mode of systemic consolidation in Russia is pronounced, first, because of the history of corruption and privatism in the Russian legal/political system. During the years after the collapse of the Soviet Union, notably, state institutions were exposed to such intense corruption and office grabbing that they approached a condition of comprehensive debilitation, and both the general levels of state capacity and, more specifically, the ability of state agencies to penetrate into diffuse parts of society were greatly undermined by their institutional privatization (Shlapentokh 1996; Tompson 2002; Taylor 2011, p. 25). In particular, this crisis was reflected in an at times egregious loss of social confidence in formal judicial institutions (Grzymala-Busse and Luong 2002), as, given the weakness of public agencies, citizens were often inclined to seek remedies for legal problems by private means. This phenomenon was widely described as *shadow justice* or *legal nihilism* (Tikhomirov 2007). Importantly, actors at the highest levels of government perceived the proliferation of shadow justice as an acute structural problem, which limited the societal reach of the legal/political system and diluted the power of the central state.^54^ Putin expressly declared that a state not consistently governed by law is a *weak state*.^55^

One main objective of the legal reforms conducted in Russia since 2000, consequently, was to fight judicial privatism or shadow justice, and, in so doing, to use increased public access to law to stabilize public institutions. In particular, litigation was promoted to ensure that citizens were more closely articulated with the political system, and that exchanges between citizen and government took place in constitutionally controlled procedures, thus intensifying the societal authority and the basic capacity of the political system. Importantly, the re-definition of administrative litigation discussed above was originally designed as a step towards the constitutional consolidation of government:^56^ that is, it was viewed as a way to give effect to the model of justice envisaged in the Constitution (Shmaliy 2013; Serkov 2003), to ensure the entry of international norms into the legal system Ivanov (2014), to guarantee “democratic dispute resolution between the citizens and the state’ (Gordeeva 2013), and—through these achievements—to enhance the “quality of the Russian state” (Starilov 2013). Indicatively, administrative litigation is now acknowledged as a core mechanism for combating the “syndrome of lawlessness” associated with shadow justice (Starilov 2005).

Such policies have had visible effects in Russia and they have helped to establish greater formality and immediacy between citizens and the state. The basic fact that litigation has increased shows that formal legal norms have penetrated more deeply into society, and the sphere of interaction between state and citizen has become more robustly constitutionalized. Public opinion surveys have clearly indicated that, at different points in society, people are increasingly willing to resolve their problems in courts, and the penetration of law into society is rising.^57^ This process of transformation can be seen in different spheres of interaction. However, it can be seen most clearly in spheres of exchange in which regular justice previously had limited penetration, especially during the institutional collapse of the 1990s.

To illustrate this, for example, the deficiency of most state functions in the 1990s meant that Russian citizens tended to look for informal routes to resolve everyday disputes, or to resort to “criminal bosses” in order to achieve justice.^58^ This trend was most visible in monetary and property disputes, in particular in debt collection (Skoblikov 2000). Through the 1990s, both individuals and organizations made wide use of the “service” provided by criminal debt collectors, typically paying 10–20% of the total value of the debt in commission charges.^59^ However, as litigation increased and new legislative measures were put in place to regulate debt collection, fewer people chose to resort to criminal intermediaries, while more people chose courts and legal collectors to retrieve debts.^60^ Indicatively, comparative opinion polls show that in 1999–2000 more than 40% of respondents were in favor of using criminal structures for help with debt collection (Skoblikov 2001, pp. 62-63). In 2010 only 5% of respondents chose this option, while 18% of respondents have opted for the help of legally operating debt collectors (Artyushkina 2011, p. 196). Increased access to justice substantially reduced the areas of society outside the direct control of state institutions, and it clearly extended the reach of formal government bodies.

In assessing the formalization of social interaction through litigation, it is important to bear in mind that there remain significant regional variations in the volume of litigation heard by Russian courts, suggesting that informal justice is still prevalent in some regions. For example, in 2015, citizens in the Far Eastern Federal District of the Russian Federation were the most judicially active. In these regions, courts of first instance heard nearly 180 civil and administrative claims per 1000 members of the region’s population. Moreover, the highest number of claims challenging normative acts and non-normative acts is recorded in the Central Federal District. The lowest rate of overall litigation was in the North Caucasian Federal District, with 60 claims per 1000 people. This is the poorest region in Russia, where use of sharia courts is common and clan structures provide important instruments for informal conflict resolution (Tsaliev 2016). There are also regional variations in human rights consciousness and in approaches to unofficial patterns of dispute resolution. For example, early research (Sheregi 2002, p. 112) shows that residents of North Caucasus, St. Petersburg, and Povolzhskiy region are more likely to resort to bribery or “informal support of powerful people” to resolve their problems than residents of other regions. Consequently, there is no entirely uniform tendency towards expansion of formal law across all parts of Russian society.

Despite this, even such variations suggest that social agents across Russia have become more responsive to formal normative modes of socio-political articulation. Even in regions where formal litigation is less widespread, more informal dispute resolution methods are increasingly incorporated under the general umbrella of the formal legal system.^61^ For example, decisions of sharia courts, which, in Russia, pertain to the sphere of informal justice, are subject to appeal before federal courts if the parties agree to present the sharia court decision as a result of mediation.^62^ In this way, sharia courts are transformed into a part of the formal legal order (Syukiyanen 2014). Further, informal legal practices now only predominate in regions where informal justice is marked by strong cultural institutionalization. For example, in the Chechen Republic unofficial petitions to the president of the Republic remain the dominant mode of dispute resolution.^63^ Even in such regions, in fact, mediation practices and reconciliation commissions created by the President of Chechnya have gradually replaced blood feuds and other means of informal dispute resolution.^64^ Moreover, the number of Chechen residents using the federal judicial system in the Republic has increased.^65^ This trend has become particularly pronounced since 2003, when full-time judicial bodies were established in the Republic.^66^

In these different ways, the reforms promoting access to the justice system have extended the normative foundations of the governmental order, so that, in different functional and regional domains, agents across society enter a more immediate relation to the government through litigation. Central to this phenomenon is the fact that litigation is used as a remedy for specific systemic weaknesses: namely, for low societal penetration of formal institutions. The growth of litigation thus results from systemic policies designed to trigger litigation, through which the use of law is expected to imprint a formal grammar on traditionally unregulated spheres of social exchange.

## Systemic consolidation 2: centre-periphery linkage

Litigation has acquired a distinctive systemic importance in Russia, further, because of the fragile center-region linkages in Russian society, which historically jeopardized the integrity of the political system as a whole.^67^ This problem was of course originally concentrated around inter-ethnic conflicts, which became dramatically manifest during the dissolution of the Soviet Union, in which, beginning in 1989, the separatist ambitions of the constituent entities of the Soviet Union proved a daunting challenge for the government.^68^ Ethnic conflicts then remained destabilizing after 1991, and they were intensified by Yeltsin’s intermittent policies of encouraging administrative entities within the RSFSR to seek separate sovereignty.^69^ From 1991 on, acute centrifugalism continued to threaten governmental cohesion, and many regions further intensified their autonomous powers,^70^ often in contravention of the provisions for autonomy contained in the formal text of the Russian Constitution.^71^ This was exacerbated by the fact that, under the Soviet system, Russian citizenship had been weakly institutionalized, and a specifically Russian citizenship, able to link different sub-units of the state to central institutions and to withstand the force of nationally intensified citizenship emerging in the regions, had not developed.^72^ However, such systemic instabilities in Russia are also evident in more generalized problems of articulation between state and society. As mentioned, the political system in Russia is afflicted by a long history of low societal penetration. One particular symptom of this is that the force of formal governance institutions recedes rapidly outside major political centers, and local actors can easily build up independent power structures. Historically, this has meant, at different times, that political institutions have only been precariously embedded in society, and they have often relied on localized patterns of patrimonial resource distribution to secure support.^73^ Arguably, in fact, this has meant that the political system as a whole is not fully nationalized and that it lacks national integrative power.

In this context, litigation has assumed core capacity-building importance in Russia, and access to law has been promoted for purposes of systemic reinforcement, in order to cement uniform foundations for government in different parts of society (Kahn et al. 2009, p. 330; Hanson and Teague 2005, p. 657; Sakwa 2008, p. 187; Easter 2008, p. 203; Yakovlev 2006, p. 1033). Historically, states that encountered powerful inner-societal enclaves of power and weak center-periphery linkage have normally overcome this, if at all, through *national democratic politicization*. That is, such states have weakened embedded centrifugal forces through a deepening of competitive democracy and participatory citizenship at a national level, usually through the solidification of political parties and correlated institutional infrastructure, which erode the basis of influence enjoyed by local actors.^74^ In Russia, a number of political measures have been used to augment the centralization of national society around the political system. These measures include central control of the appointment of the regional governors, with appointments linked to promotion of party interests.^75^ These measures also include conventional use of patronage and patrimonial methods for securing local and regional support for the government.^76^ These measures have also included the creation of a central governing party, *United Russia*. Clearly, however, the democratic dimensions of government have not been comprehensively reinforced under Putin, and a strong, integrative party system has not materialized.^77^ As a result, classical openings for exercise of national citizenship rights, although not absent, are constrained. Under these conditions, litigation is promoted as an important means of political-systemic nationalization, and one reason for the stimulation of litigation is that it is seen to compensate for the weak institutionalization of citizenship practices in other spheres. Whereas other techniques used to promote the nationalization of Russian society are at least partly authoritarian, litigation contains a strong participatory dimension, and it forms a functional equivalent to more standard democratic citizenship practices. In this respect, the nationalizing function of political citizenship is displaced into the legal system. Indeed, Putin’s policies of judicial reform were specifically designed with this end in mind. These policies were intended to create a *single legal space* across the entire Russian Federation, and to impose the formal law of the Constitution across all parts of society.^78^ Importantly, in his first annual address to the Federal Council in 2000, Putin declared that it was “intolerable” for subjects of the Federation to act in contravention of the Constitution.^79^ Accordingly, litigation has been formally identified as a means for tightening center-periphery linkages and remedying weak nationalization in Russia, and the growing volume of litigation has provided some remedy for historic problems of state diffuseness.

The impact of litigation on center-periphery relations results, first, from the general structure of the Russian judicial system, which is strictly centralized, and tends to heighten the cohesion of the political system as a whole. All Russian courts, including justices of the peace are funded from the federal budget.^80^ All federal judges are appointed by the President,^81^ and justices of the peace are appointed or elected by regional parliaments.^82^ Moreover, one important function of the Supreme Court is to ensure uniform application of federal law by all judges across the country. Through its systematization of court practice and plenary resolutions, the Court formulates guiding principles that, although not classified as sources of law, form compelling directives for judges. Maintaining consistency in judicial practice is thus understood as a common obligation for all members of the judiciary, regardless of their regional location.^83^ The result of this judicial unity is that litigation at any level of society necessarily establishes a deeper normative linkage between central authorities and peripheral regional zones, and it connects all parts of society immediately to the political system and the Constitution.

This centralizing impact of litigation is particularly visible in the number of appeals considered by higher-instance courts, in which higher courts have overturned lower-court rulings. Rescinding of lower-court rulings in higher courts can be seen as a key component in the construction of a uniform legal system, and it forms a final nexus in which litigation triggers intensified intersection between peripheral parts of society and the core political system. On average, appellate instances in Russia overrule or alter up to 14% of appealed decisions of local justices of the peace, and they overturn around 13% of appealed decisions of first instance courts. The highest rate of appeals against justices of the peace is seen in Privolzhskiy and Central Federal Districts, while the lowest is seen in the North Caucasian Federal District. However, the picture is reversed when it comes to the outcomes of appeals. The North Caucasian and Far Eastern Districts show the highest rate (16 and 17%) of appealed decisions of justices of the peace that are overturned or altered by federal courts. Similarly, the North Caucasian, Far Eastern, and Crimean Federal Districts have the highest percentage (respectively 20%, 15%, and 25%) of appealed decisions of federal courts that are annulled or altered by higher-instance courts. Therefore, appeals are most successful in regions where informal legal practices are most prevalent. Regional variations in appeal statistics indicate, consequently, that measures for ensuring legal unity are at least partially effective.

The impact of litigation on centre-periphery relations results, second, from the fact that administrative litigation, in particular, brings regional agencies more firmly under the control of central government. Notable in this regard, in fact, is that the CACP has declared that nation-wide access to administrative justice is one of the priorities of the government’s policy.^84^ The CPC, which previously regulated administrative litigation, did not have this objective, and the 2002 *Arbitrazh* Procedure Code only presumed accessibility of justice in economic activities.^85^ The CACP, however, is expressly intended to augment the consistency of law, through litigation, as part of a strategy of systemic nationalization. Indeed, in 2015 special measures were introduced to promote uniform application of the new administrative litigation regulations throughout the country. For example, the Supreme Court issued an authoritative commentary on the CACP, explaining how it should be applied by all Russian courts.^86^ In 2015, the Supreme Court provided a summary of relevant judicial practice, which was designed to simplify the transition to the new litigation procedures.^87^ These summaries were later replicated in explanatory notes issued by regional courts (such as *krayevye sudy*, *oblastnye sudy*) and made accessible on course websites for legal professionals and the general public.^88^ Such commentaries are not considered sources of law, but they are generally accepted as instruments of judicial policy (Artemova and Lichko 2014). Above all, these commentaries are designed to enhance the consistency of litigation outcomes, and, accordingly, to lead to increased trust in the judiciary (Yaroslavtsev and Yaroslavtseva 2012).

It is still too early to assess the impact of the CACP on actual litigation. However, it is possible, tentatively, to suggest that it extends the reach of the national legal/political system. The official statistics on administrative litigation show that since adoption of the CACP in 2015 courts in most regions have employed the code. This is especially noteworthy in judicial review cases, in which, as they are conducted by courts supervising regional administrative law makers, the nationalizing impact of procedural reforms is most manifest. Since 2015, the courts of 60 federal subjects have heard administrative-law cases involving review of regional legislation.^89^ Of over 570 cases of this type 342 were first-instance cases and 230 were appellate cases. The leading region in first-instance cases is Primorskiy Krai (Vladivistok), with nearly seventy cases. Next come Moscow Region and Perm Krai in Central Russia (22 and 21 cases), followed by more remote Novosibirsk Region, Sevastopol, Yakutia Republic, Altai Krai, and Khabarovskiy Krai (around fifteen cases on average). The majority of central Russian regions heard two-to-eight cases.

Overall, administrative litigation appears to be acquiring diffuse impact across Russian society, and the use of the CACP to initiate litigation is not regionally confined. As with the partial eradication of shadow justice, rising litigation, and widening access to law can be seen to embed a set of practices in society that formalize relations between center and periphery and harden the national penetration of the political system. This can again be observed as a phenomenon, in which the government deliberately stimulates demand for law in order to connect national society more consistently to the governmental order. Indeed, litigation and appeal processes play an important role in tightening the legal relation between center and periphery, and effectively in *constitutionalizing* interactions across national society as a whole. In this respect, litigation clearly results from systemic pressures that impact political actors, linked to low institutional capacity, and it reflects a *strategic policy of stimulating demand for law*.

## Litigation and constitutional outcomes 2

In the above respects, rising litigation is attributable to distinct causes in the Russian polity. Whereas rising litigation is usually explained as the result of anti-systemic behavioral patterns, in Russia litigation is clearly induced by public policy. Nonetheless, as in other polities, we can also observe that, once it is stimulated, litigation cannot be proportioned solely to governmental objectives, and it acquires a certain independent norm-producing force, often generating legal principles and political outcomes in quite contingent fashion. In some ways, the fact that litigation is promoted to imprint a constitutional grammar on interactions between citizen and state means that citizens are cast, necessarily, as constitutionally implicated legal agents. As a result, interactions between citizen and state increasingly generate public obligations, which bear directly, and at times in normatively transformative fashion, upon government agencies themselves. Russian citizens, in other words, are encouraged to litigate for systemic reasons. In this process, however, they are inevitably constructed as constitutionally formative actors, and, as in other polities, acts of litigation assume an important role in defining constitutional norms for the polity. The fact that political actors rely on litigation for systemic consolidation at times creates unusual, unintended patterns of counter-systemic norm production. Notably, the systemic function of litigation strengthenms leads to a relative strengthening of legal rights in a general sense. As discussed, this is reflected in the hardening of procedural rights regarding access to justice and of protective rights against administrative violation, and it entails assimilation of international norms into domestic law. However, this also means that rights can assume more volatile normative force and they can give rise to less managed constitutional effects.

### Strategic litigation

The transformative, norm-producing quality of litigation in Russia is visible, first, in relatively typical processes of strategic litigation, which have clear similarities with cases in other countries.

Although academic literature, both in Russian and in English, addressing standard patterns of strategic litigation in Russia is very scarce, it is clearly documented that this is a topic of increasing importance, and there exist openings for strategic legal mobilization (Kachanov 2008). Guidance for lawyers working in the area of strategic litigation is usually provided by foreign NGOs.^90^ Such guidance is then adopted by progressive human rights litigants in Russia, who then publish their own guidelines for litigation. The most famous and successful strategic litigant is the NGO “*Sutyajnik”* (literally meaning “litigant” in Russian) in Ekaterinburg. For more than twenty years, the lawyers of this regional organization have trained judges in the application of international human rights law, and, among other activities, they have filed strategic cases related to discrimination, freedom of assembly, barriers for NGO work, violence in detention, and other “priority areas.”^91^ Importantly, Russian “strategic” lawyers use a wide range of working methods, and they promote judicial review, direct use of constitutional and international norms, and, in particular, reference to the ECHR and to ECtHR jurisprudence to reach transformative legal objectives. They also file applications to the Russian Constitutional Court (RCC) and the ECtHR. Often, alongside administrative cases that are directly aimed at challenging federal, regional, or local policies in the defense of public interests, strategic lawyers use “private interest” cases to achieve strategic goals of combating structural problems in the legal/political system. Such mechanisms are deployed in the areas of consumer protection, employment law, or environmental law.^92^ Prominent strategic cases won by the *Sutyajnik* lawyers in Russian courts and in the ECtHR concern violation of fair trial procedures,^93^ illegal forced detention in a psychiatric hospital,^94^ and violation of fair election procedures.^95^

One very notable success story of the *Sutyajnik* lawyers is a set of cases challenging the ban on long prison visits for persons serving a life sentence. In these cases, the lawyers used relevant ECtHR jurisprudence, to induce significant legislative changes.^96^ First, the lawyers obtained the result that the RCC invalidated the ban,^97^ and, in fact, the RCC reversed its own previous decisions on the same question.^98^ Importantly, the RCC used the principle of *the best available protection* in deciding whether to apply national or international regulations, and it concluded that ECtHR jurisprudence concerning the family rights of prisoners, providing higher protection for human rights, should prevail. In so doing, the Court invalidated the respective norm of the Russian Prison Code, making express reference to Article 8 ECHR in its interpretation by the ECtHR.^99^ Second, following the RCC ruling, a federal law was adopted to permit one long family visit per year for persons imprisoned for life.^100^ This means that more than 2000 families in the same situation have received an effective right to arrange prison visits.^101^ Strategic litigation thus shapes sensitive areas of public policy, and its outcomes are partly determined by international law. In such respects, strategic litigation in Russia is close to the model of contentious norm formation documented in other polities.^102^

### Hardening norms of public law

The transformative, norm-producing impact of litigation in Russia is generally visible in the fact that litigation often reinforces the authority of constitutional norms, and it leads to the expansion of constitutional rights when such rights are constructed in conjunction with international law. In this way, even when it is motivated by purposes of social control, litigation forms a constitutional impetus in society, with far-reaching impact on the political system.

After the collapse of the Soviet Union, notably, Russia inherited a very patchy system of legal regulation, in which legal certainty was limited. This problem was particularly acute in procedural legislation referring to civil and administrative litigation. Prior to the adoption of the new procedural codes, consequently, judges were encouraged to apply the Constitution in any case in which a gap in regulation was detected.^103^ This practice meant that the courts became accustomed to independent decision-making in cases of uncertainty, and they promoted constructive interpretation of constitutional law. This fact has particular importance for recent developments in administrative litigation. Notably, as administrative litigation has increased over recent years, judges have continued their practice of applying constitutional norms directly, and the rising quantity of litigation has acted as a catalyst for enhanced judicial materialization of constitutional norms. In fact, courts have shown surprising willingness to curtail the powers of government agents in sensitive cases, and even to use constitutional authority to tackle the decisions of the political branch.

The willingness of courts to expand constitutional law is exemplified, for instance, by cases in which Russian courts have applied human rights norms to challenge federal immigration policy, especially concerning deportation of aliens. The courts have done this by insisting that immigration policies must show regard for the family ties, health conditions, and risks to the life of persons subject to deportation by public officials.^104^ The Supreme Court summarized judicial practice in this regard in its 2013 guidelines, advising lower courts to take Article 8 ECHR into consideration in all cases concerning administrative deportation of foreign citizens.^105^ Furthermore, the willingness of courts to expand constitutional law is exemplified by cases in which the RCC has intervened in questions regarding taxation policy, a domain traditionally reserved exclusively for governmental decision makers. In the period 2007–2014, the Court invalidated several provisions of the federal Tax Code.^106^ As a result of some of these cases, important aspects of taxation policy were amended. These changes entailed the elimination of double taxation for individuals,^107^ the protection of the right to file applications for claims against the federal taxation authorities,^108^ and protection of the rights of businessmen against excessive inspection.^109^ In cases of legal uncertainty, moreover, courts have continued to apply international norms even if it places additional restrictions on public agencies. For example, in February 2017 a district court in Voronezh reached an unpopular decision on the basis of the Constitution and international law. The court declared legal a protest against the war in Syria and against lack of direct elections in the appointment of the city’s mayor. In this case, the court deemed the demonstration lawful, noting that the public meeting had been agreed with the local authorities, while the measures used to prevent the protest did not constitute a legitimate and proportionate limitation of the constitutional freedom of assembly. Importantly, in support of this conclusion, the district court referred to Article 11 ECHR and the ECtHR jurisprudence.^110^

In such respects, litigation is not simply a controlled practice. In fact, governmental stimulation of litigation of itself creates a counter-systemic impulse, and litigation also generates robust, binding norms for the political system itself. The fact that litigation is encouraged to promote systemic consolidation necessarily contributes to a logic of political transformation.

### Autonomy of the judicial system

The norm-producing impact of litigation in Russia can also be identified within the legal system itself, and it has led, discernibly, to an increase in the basic autonomy of the judicial system as a branch of government. Notably, the rising volume of litigation heard by the courts has involved an increasing use of the human rights provisions expressed in the Constitution to establish violations and to provide remedies. In turn, this has led to a growing reliance of the courts on international human rights law to develop their jurisprudence. Through this, the courts have cemented and intensified their own position, within the governance system as a whole, as relatively independent producers of norms with constitutional implications.

First, the growing autonomy of the judiciary is evident in the fact that the courts increasingly apply legal concepts derived from ECtHR jurisprudence (Starzhenetskiy 2013). To be clear, the reception of ECtHR in different regional courts is not uniform, and it sometimes requires significant effort on the part of strategic litigants.^111^ Moreover, the RCC has recently gained some notoriety for ruling, like Constitutional Courts or superior courts in other European countries, that the national Constitution has supremacy over conflicting judgments of international courts and tribunals.^112^ Nonetheless, use of the ECHR by the courts is not generally restrictive, and the ECHR often has significant constitutional implications in Russia.

An important example of the constitutional outcomes of rulings based in the ECHR can be seen in the fact that the courts follow ECtHR precedents in imputing liability for public acts. Notably, a 2009 ruling of the Supreme Court Plenum and a 2012 Constitutional Court Ruling both used international law to expand the scope of responsibility for agents performing public functions, insisting that private organizations with a special public status could be subject to standard norms of public liability.^113^ In 2013, this principle was solidified in a federal law.^114^

A further important example of the constitutional impact of the ECHR is evident in the fact that judicial use of ECtHR jurisprudence has led to a wide acceptance of the doctrine of proportionality in Russian courts.^115^ In 2013, the Supreme Court established a clear-cut general rule on the use of proportionality principles based in the ECHR, stating that “limitation of rights and freedoms is only allowed if there are relevant and sufficient grounds for such a restriction.”^116^ The use of proportionality is particularly important as evidence of the autonomous norm-producing impact of litigation, as it substantially elevates the role of the courts within the polity, and it allows courts to construct public-legal norms in independent fashion. For example, in 2012, the Court issued an important ruling on the unconstitutionality of legislation restricting the legal position of persons with limited legal capacity due to mental illness and other similar causes.^117^ In this ruling, the Court appealed to the principle of proportionality in order to provide a nuanced approach to the civil rights of people experiencing mental-health challenges. As a result, amendments were introduced to Arts 29–32 of the Civil Code, which meant that judges were allowed to determine limitations to the rights of persons with mental health problems in accordance with the actual state of the particular person concerned.^118^ In such cases, the courts have applied proportionality reasoning, based in the ECHR, to scrutinize the substantial content and impacts of laws, and to prescribe generally binding principles for all legislation (see Rivers 2006; Sweet and Mathews 2008). In consequence, the norm-producing power of courts increased substantially.

Seond, the growing autonomy of the judiciary is manifest in the fact that increasing reference to international law has reinforced the role of judicial precedent in the Russian legal system, and it has partly converted the legal order into a precedent-based system.^119^ Indeed, there is evidence that judges are changing their approach from one based in their traditional mechanical reproduction of legislation to a more comparative approach, showing acceptance of judicial precedent as a means to promote consistency and stability in the legal system.^120^ Although the authority of precedents is not uncontested,^121^ the need to accommodate ECtHR judgments within the Russian legal system has led to the increasing recognition that some judgements assume near-precedential force (Granat 1998; Zverev 2006). It was strongly promoted by Anton Ivanov, the Chairman of the Higher Arbitrazh Court that was abolished and merged with the Supreme Court in 2014.^122^ According to established practice, the guiding principles and rulings of the Supreme Court possess greater weight than decisions of lower courts.This trend is particularly visible in the work of *arbitrazh* courts. However, in more recent practice, it has become common for courts to refer to the decisions of courts that are located at the same level in other regions.^123^ To be sure, such reference is merely informative and does not imply that judicial decisions have binding force as would be the case in the common-law doctrine of precedent. Nonetheless, precedent is becoming de facto a practical part of the Russian judicial system, and courts increasingly rely on precedents, both of international extraction and established purely under domestic law.

One result of the increasing use of precedent is that litigation instills uniformity within the legal system. Indeed, as court findings are increasingly based either in ECHR norms or in precedent, the judiciary becomes increasingly reliant on inner-legally constructed norms for the production of rulings. One further result of this is that the autonomy of the judiciary as a norm-provider is reinforced. Indeed, as with the growth of proportionality, the rising use of precedent means that actors in the legal system are able to generate powerfully pervasive norms.^124^ As a result, judicial rulings construct a normative framework for public functions that is relatively independent of any obvious policy decision.

Overall, on these counts, litigation has acquired a pervasive norm-producing, even constitutional impact in Russia. In a range of ways, the growth of legal action has triggered constitutional reactions within the legal system, which have a broad impact on the exercise of government power. On one hand, as discussed, the growing volume of litigation is induced by systemic motives, and it is promoted as a means to harden the effective authority of the state. However, this systemic process also contains transformative elements, and it produces normative structures with a real constitutional valence, strong enough to formalize a distinct system of norms for the exercise of governmental powers. In this respect, the patterns of litigation that can be observed in Russia reflect a constitutional hybrid, combining inner-systemic and counter-systemic results. Litigation assumes constitutional functions quite specific to a hybrid state, in which the government promotes use of law for systemic consolidation, aimed to increase social penetration of state agencies. Yet, the government is also forced to accept the *counter-power*, expressed in the process of normative, even constitutional transformation, to which such consolidation gives rise.

Unlike other societies, the constitutional force of litigation in Russia is not necessarily expressed through strategic legal activism. Indeed, when we examine the politics of litigation in Russia, we do not look primarily for highly mobilized collective agents’ intent on identifying or exploiting new legal opportunities for strategic ends. Instead, we look, mainly, for a diverse array of litigants, availing themselves of legal procedures opened to them by the government, often filing suits with implications for basic human rights. Often, in fact, such agents *may not perceive themselves* as mobilized, and they may not understand themselves as implicated in citizenship practices. However, litigation in Russia engenders political outcomes whose consequences clearly parallel those evident in less controlled jurisdictions. Even where shaped by systemic prerogatives, litigation widely leads to transformative outcomes or even to outcomes that re-orient constitutional law.

## Conclusion

Public-law litigation in Russia, especially as it addresses human rights law, is not widely researched, but it is an important phenomenon. In Russia, litigation is deliberately triggered by government agencies because the government relies on litigation for state capacity building and political-systemic nationalization. As a result, however, litigation also assumes a certain norm-constitutive autonomy, and systemic stimulation of litigation has consequences that are not fully controllable. Naturally, this does not imply that judicial reform policies have created a comprehensive accountability structure for the Russian government. It is clear that, in parallel to these reforms, the years since 2000 have witnessed a process sometimes described as a ‘hyper-strengthening’ of presidential power in Russia (Abdrakhmanov 2012). Even more alarmingly, recent years have witnessed an increasing politicization of criminal justice.^125^ To this degree, there is growing evidence to sustain the common assertion that much governmental practice occurs in a para-constitutional sphere.^126^ However, the reinforcement of constitutional norms through litigation is also a fact of legal/political life in Russia, and it forms part of the complex picture of a hybrid polity. A distinctive sociological framework, relativizing the common claim that public-law litigation has a contentious character, is required to capture this.

Notable in this respect is the fact that the simultaneously systemic/counter-systemic status of litigation in Russia is not of an absolutely sui-generis nature. Manifestly, Russia has a distinctive position in any cross-national comparison concerning litigation and legal mobilization. In Russia, first, litigation is at once more systemically controlled and less overtly politicized than in other countries. Second, litigation occurs in an institutional setting in which organs of political mobilization are not galvanized by inter-party competition. Third, the starting point for development of state capacity after the crises of the 1990s was particularly low. On the latter two counts, litigation in Russia assumes unusually extensive capacity-building functions. In other respects, however, litigation in Russia, especially in matters referring to human rights, throws distinctive light on the consequences of contentious litigation in other societies. Using Russia as an extreme example, we can see that litigation with implications for human rights widely reflects a dialectical relation between litigation and the governance system, and, even where it involves acts of anti-government mobilization, it frequently has systemically beneficial outcomes, especially regarding state capacity building and systemic nationalization.

An example of this can be seen in contemporary Colombia, where litigation regarding human rights law is strategically encouraged as a means to build state institutions. In fact, the Constitutional Court in Bogotá has formulated this policy in “Weberian terms,” arguing that promotion of human rights litigation enables the state as a whole to demonstrate legitimacy by “monopolizing the exercise of force” in society.^127^ A further version of this can be seen in India, where litigation is incentivized as a means to impose cohesion on a society marked by extreme regional and class divisions.^128^ In some respects, however, the case most clearly illuminated by comparison with Russia is the United States during the longer civil rights era, especially in the 1960s. The counter-systemic dimension of legal mobilization was more pronounced in the United States in the 1960s than in contemporary Russia. However, legal mobilization was clearly encouraged inner-systemically, notably by the broad construction of civil rights proposed by the Supreme Court.^129^ More importantly, in both settings, rights-related litigation acted as a means both for reinforcing state capacity building for enhancing political-systemic nationalization.^130^ Indeed, in the United States in the 1960s and contemporary Russia, the fact that litigation led to domestic enactment of human rights norms projected in the international arena assumed core importance in a process of systemic nationalization.^131^ This aspect of civil-rights litigation in the United States has been perceived in relevant literature, but it has not been constructed as a broad sociological phenomenon. In this regard, Russia can be seen as an extreme example of a state which relies on litigation for systemic capacity building and nationalization, and therefore tolerates the build-up of normative counter-power that this necessitates. However, we can also cite Russia as a paradigm, which offers a general framework for interpreting legal mobilization. The fact that litigation institutionalizes patterns of citizenship that extend the reach of the political system into national society might be a reason, quite generally, why governments are prepared to accept strategic litigation. Other commentators have noted, accurately, how governments with some authoritarian propensities may encourage high-level access to court, and they have stressed the symbolic gains that such governments obtain from this in signalling their legitimacy (Ginsburg 2003). The claim can be added to analysis of courts in hybrid systems, however, that governments may well also identify systemic nationalization, enhancing their own institutional capacity, as an unintended beneficial result of public-law litigation, and this may also have a value that outweighs its political inconveniences.

## Footnotes

1 See the seminal perspective in Black (1973). See for general perspectives Scheingold (1974); Zemans (1983); McCann (1986; 2004); Epp (1998); Kelemen (2003). For an overview see McCann (2008).

2 For a very high appreciation of the role of litigants in ending discrimination in the United States see Francis (2014).

3 For studies of litigation as mobilization for more particular rights see (with a focus on Canada and the UK) Vanhala (2011); Burstein (1991); McCann (1994); Feeley and Rubin (1998); Epp (2009). See for more sceptical comment Rosenberg (1991); Brown-Nagin (2005).

4 For views which stress the role of litigation as a distinctive expression of citizenship, see Siegel (2006); Eskridge (2001); Yeazell (2004).

5 See this claim in Sabel and Simon (2004); Epp (2009, p. 24). See an extreme version of this claim in Hunt (1990).

6 This claim is clear in Epp (1998, p. 24). This is still more emphatically stated in Rubin and Feeley (2002).

7 For sociological antecedents see Tarrow (1994); Nettl (1967).

8 See Ledeneva (2008); Mazmanyan (2015).

9 For an exception see Vadim Volkov, ‘Zhaloba kak mekhanizm mobilizatsii zakona: kak poddelnyye “zhaloby” podrazhayut deyatelnosti grazhdanskogo obshchestva i legitimizatsii ispolneniya” (Complaint as a Mechanism to Mobilize the Law: How Fake “complaints” Mimic the Activity of Civil Society and Legitimize Enforcement) *Vedomosti*, 14 December 2016, available from: http://www.vedomosti.ru/opinion/articles/2016/12/14/669681-donos-mehanizm-mobilizatsii. All translations from Russian into English are by Maria Smirnova.

10 See Hendley (2013); Trochev (2012).

11 See the discussion below. See also Hendley (2011).

12 All statistical data in this article, unless specifically mentioned otherwise, is extracted from the official database of judicial statistics administered by the Judicial Department of the Supreme Court of the Russian Federation, available from: http://www.cdep.ru/index.php?id=79

13 See Reuter (2010)

14 Schedler (2006, p. 4). Measured by two standard definitions of democracy, those proposed by Dahl and Tilly, Russia would have to be classified as a hybrid. Russia probably does not meet any point in Dahl’s 7-criteria measurement of democracy. But none of the criteria is entirely absent. See Dahl (1989). Tilly sees democracy as involving ‘broad, equal, protected, binding consultation of citizens with respect to state actions’ (Tilly 2007, p. 34), and as presupposing ‘broad citizenship, equal citizenship’, and ‘protection of citizens from arbitrary action by government officials’ (Tilly 2000, p. 4). Tilly’s definition does not map directly onto political realities in Russia, but some of its components are in evidence. Following more recent definitions of democracy, for example, that set out by Munck, Russia would probably be classified as predominantly authoritarian, owing to weak electoral competition. See Munck (2016). In definitions which accentuate the importance of legal order in a democracy, Russia would appear as only partly deviant from a standard norm. See for example Bogaards (2009). For other accounts of Russia as a hybrid polity see Robertson (2011); Petrov et al. (2014); Lauth (2015).

15 One leading article on this question sets out a typology of regime authoritarianism, which identifies a deficient legal system as a key characteristic of an authoritarian polity. Such a system is one in which the rule of law is challenged by alternative normative systems or marked by high acceptance of alternative norms (Lauth and Sehring 2009). Russia may show these characteristics in weakened form, but it is also marked by intensified commitment to judicial integrity.

16 Interpretations that compare Putin’s system with earlier models of authoritarianism show only limited understanding of the relation between state organs in fully authoritarian states. In fascist states, with which Putin is compared, the construction of independent patterns of judicial norm formation was impossible. See rather a questionable account of Putin’s legal order as an example of classical authoritarianism in Sakwa (2010). See the simplistic invective in Motyl (2016). The claim that Russia is a fascist state is simply absurd. The definitional proto-type fascist state, Italy under Mussolini, was designed so as to minimize judicial autonomy. Leading legal theorists of Mussolini’s regime argued that *judicial power* was to be used as a vertical medium of social control. See Panunzio (1933, p. 31).

17 See Pedriana and Stryker (2004). In discussing state capacity, we accept the idea that this refers to the growth of the ‘civil administrative capacities’ of the state (Skocpol and Finegold 1982, p. 271). We claim, though, that expansion of state capacity presupposes a normative/constitutional structure, in which state functions penetrate deeply and inclusively into national society. We link this normative dimension of state capacity to litigation and its promotion.

18 On this phenomenon in Soviet era see Devlin (1995, p. 38); Easter (1996).

19 President of the Russian Federation Decree No. 2334 of 31 December 1993 ‘O dopolnitelnykh garantiyakh prav grazhdan na informatsiyu’ (On Additional Guarantees of the Rights of Citizens to Information).

20 Russian National Network of Public Centres for Legal Information, available from: http://www.pcpi.ru/.

21 All procedural codes were adopted during the early years of Putin’s presidency: Grazhdanskiy protsessualniy kodeks Rossiyskoy Federatsii (Civil Procedure Code) No. 138-FZ of 14 November 2002; Arbitrazhniy protsessualniy kodeks Rossiyskoy Federatsii (Arbitrazh Procedure Code) No. 95-FZ of 24 July 2002; Kodeks Rossiyskoy Federatsii ob administrativnykh pravonarusheniyakh (Code of Administrative Offenses) No. 195-FZ of 30 December 2001.

22 Since 2004 all fees for all legal actions are consolidated in one document, the Tax Code (Nalogoviy kodeks), Chapter 25.3 of the Second Part of the Code, as introduced by the Federal Law No. 127-FZ of 2 November 2004. The court fee is now established as a firm figure, 100 roubles ($2 in 2004) for claims initiated by individuals. In more than 10 years this amount has only risen slightly, to 300 roubles.

23 On litigation and opportunity structures see Wilson and Cordero (2006); Kapczynski (2008); Vanhala (2012).

24 Government of the Russian Federation Decrees No. 805 of 20 January 2001 ‘O federalnoy tselevoy programme “Razvitiye sudebnoy sistemy Rossii” na 2002–2006 gody’ (On the Federal Target Program ‘Development of the Russian Judicial System’ in 2002–2006); No. 583 of 21 September 2006 ‘O federalnoy tselevoy programme “Razvitiye sudebnoy sistemy Rossii” na 2007–2012 gody’ (On the Federal Target Program ‘Development of the Russian Judicial System’ in 2007–2012); No. 1406 of 27 December 2012 ‘O federalnoy tselevoy programme “Razvitiye sudebnoy sistemy Rossii” na 2013–2020 gody’ (On the Federal Target Program ‘Development of the Russian Judicial System’ in 2013–2020).

25 Federal Law No. 262-FZ of 22 December 2009 ‘Ob obespechenii dostupa k informatsii o deyatelnosti sudov v Rossiyskoy Federatsii’ (On Ensuring Access to Information about Activities of Courts in the Russian Federation).

26 Supreme Court of the Russian Federation Plenum Ruling No. 35 of 13 December 2012 ‘Ob otkrytosti i glasnosti sudoproizvodstva i o dostupe k informatsii o deyatelnosti sudov’ (On the Openness and Transparency of Judicial Proceedings and Access to Information on the Activities of Courts).

27 See Article 4 of the Federal Constitutional Law No. 1-FKZ of 31 December 1996 ‘O sudebnoy sisteme Rossiyskoy Federatsii’ (On the judicial system of the Russian Federation).

28 Poslaniye Prezidenta Rossiyskoy Federatsii Federalnomu Sobraniyu ‘Rossii nado byt silnoy i konkurentosposobnoy’ (President of the Russian Federation Annual Address to the Federal Council ‘Russia Needs to be Strong and Competitive) *Rossiyskaya Gazeta* 71, 19 April 2002.

29 Poslaniye Prezidenta Rossiyskoy Federatsii Federalnomu Sobraniyu (President of the Russian Federation Annual Address to the Federal Council) *Rossiyskaya Gazeta* 93, 17 May 2003.

30 Kodeks administrativnogo sudoproizvodstva Rossiyskoy Federatsii (Code of Administrative Proceedings of the Russian Federation) No. 21-FZ of 8 March 2015.

31 Explanatory note to the draft federal law No. 381232–4 (Code of Administrative Proceedings), available from: http://asozd2.duma.gov.ru/main.nsf/(Spravka)?OpenAgent&RN = 381,232-4

32 Art 10(1)(7), Federal Law No. 212-FZ of 21 July 2014 ‘Ob osnovakh obshchestvennogo kontrolya v Rossiyskoy Federatsii’ (On the basics of citizens’ control).

33 Supreme Court Plenary Rulings No. 5 of 10 October 2003 ‘O primenenii sudami obshchey yurisdiktsii obshchepriznannykh printsipov i norm mezhdunarodnogo prava i mezhdunarodnykh dogovorov Rossiyskoy Federatsii’ (On application by courts of general jurisdiction of universally recognised principles and norms of international law and international treaties of the Russian Federation); No. 21 of 27 June 2013 ‘O primenenii sudami obshchey yurisdiktsii Konventsii o zashchite prav cheloveka i osnovnykh svobod ot 4 noyabrya 1950 goda i Protokolov k ney’ (On application by courts of general jurisdiction of the Convention for Protection of Human Rights and Fundamental Freedoms of 4 November 1950 and the Protocols thereto); No. 23 of 19 December 2003 ‘O sudebnom reshenii’ (On judicial decision).

34 Article 11(1) of the Civil Procedure Code; Article 13(1) of the *Arbitrazh* Procedure Code; Article 15(1) of the Code of Administrative Proceedings.

35 In 2007 the Supreme Court applied Art 6 ECHR to overturn decisions of lower courts as violating the principle of legal certainty in matters of substantive law (provision of pension). See Supreme Court Ruling No. 6-V07–28 of 2 November 2007. See in more detail on application of Art 6 Burkov (2010a).

36 See Supreme Court Plenary Rulings No. 8 of 29 May 2014 ‘O praktike primeneniya sudami zakonodatel’stva o voinskoy obyazannosti, voyennoy sluzhbe i statuse voyennosluzhashchikh’ (On the practice of application by courts of legislation on military duty and military service and the status of servicemen); No. 41 of 19 December 2013 ‘O praktike primeneniya sudami zakonodatelstva o merakh presecheniya v vide zaklyucheniya pod strazhu, domashnego aresta i zaloga’ (On the practice of application by courts of legislation on preventive measures in the form of detention, house arrest and bail).

37 For example, following the pilot judgment *Burdov v. Russia (No. 2)* of 15 January 2009 new federal legislation was adopted to provide compensation for lengthy trials. The same guarantee was reproduced in the Code of Administrative Proceedings. See Federal Law No. 68-FZ of 30 April 2010 ‘O kompensatsii za narusheniye prava na sudoproizvodstvo v razumnyy srok ili prava na ispolneniye sudebnogo akta v razumnyy srok’ (On compensation for violation of the right to justice in reasonable time or the right to execution of the judgment in reasonable time).

38 See Federal Law No. 262-FZ of 22 December 2009 ‘Ob obespechenii dostupa k informatsii o deyatelnosti sudov v Rossiyskoy Federatsii’ (On ensuring access to information about activities of courts in the Russian Federation). Internal regulations of the Supreme Court (in force 2009–2011) mentioned that the Supreme Court ‘in cooperation with the Council of Europe develops and implements joint programs aimed at further promoting judicial and legal reform in the Russian Federation and increasing efficiency and transparency of justice in Russia.’ (para. 8.2.)

39 Resolution of the VI Session of the Russian National Congress of Judges ‘O sostoyanii pravosudiya v Rossiyskoy Federatsii i perspektivakh yego sovershenstvovaniya’ (On the current state of justice in the Russian Federation and the prospects of its improvement). 2 December 2004. Available from: http://www.ssrf.ru/page/830/detail/.

40 Source of statistical information is: http://www.arbitr.ru/press-centr/news/totals/index_ar.htm. For parallel earlier analysis of these processes see Hendley (2002); Solomon (2004).

41 Kodeks Rossiyskoy Federatsii ob administrativnykh pravonarusheniyakh (Code of the Russian Federation on administrative offenses) No. 195-FZ of 30 December 2001.

42 See on this Starilov (2003); Solovieva (2001).

43 The Code of Administrative Proceedings defines ‘administrative and other cases arising from public law relations’ as ‘cases aimed at protection of violated or disputed rights, freedoms and legitimate interests of citizens’ (Art. 1(1)). Cases arising from public law relations always have a public authority on at least one of the sides of the conflict. See for analysis Tikhomirova (2011).

44 Chapter 2.1 on Pre-Judicial and Extra-Judicial process of challenging actions and decisions of public bodies providing state or municipal services was introduced into the Federal Law No. 210-FZ of 27 July 2010 ‘Ob organizatsii predostavleniya gosudarstvennykh i munitsipalnykh uslug’ (On the organization of provision of federal and municipal services) by the Federal Law No. 383-FZ of 3 December 2012.

45 These and other measures were discussed during a conference organised by the Russian University of Jurisprudence on 20 October 2015, see Mamykin & Ryabtseva (2016).

46 For example, the concept of the new Unified Civil Procedure Code approved by the State Duma on 8 December 2014 recomends the introduction of compulsory representation by a professional lawyer in all civil claims, as it is done in all criminal and, since 2015, administrative claims (para. 47.1.12 of the project).

47 In 2013 President Putin stated the need to increase the authority of private arbitration, see Annual Address of the President of the Russian Federation to the Federal Council, 12 December 2013, available from: www.kremlin.ru. Following his suggestion, a federal law was adopted in 2015 regulating the new status of private arbitration, see Federal Law No. 382-FZ of 29 December 2015 ‘Ob arbitrazhe (treteyskom razbiratelstve) v Rossiyskoy Federatsii’ (On arbitration in the Russian Federation) *Rossiyskaya Gazeta* 297, 31 December 2015.

48 In 2011 President Medvedev called for stepping up the efforts to encourage conciliation procedures in order to lower the courts’ workload, at Presidential meeting with judges, 26 July 2011, available from: https://www.1tv.ru

49 Federal Law No. 193-FZ of 27 July 2010 ‘Ob alternativnoy protsedure uregulirovaniya sporov s uchastiyem posrednika (protsedure mediatsii)’ (On alternative dispute resolution procedure involving a mediator (mediation procedure)), *Rossiyskaya Gazeta* 168, 30 July 2010.

50 Such as the Intellectual Property Court, established as an independent type of *arbitrazh* courts in 2011 and taking away at least 10,000 claims from the *arbitrazh* statistics, see Federal Law No. 4-FKZ of 6 December 2011 and *Svedeniya o rassmotrennykh arbitrazhnymi sudami Rossiyskoy Federatsii delakh, svyazannykh s zashchitoy intellektualnoy sobstvennosti, v 2012–2013 gg* (Information on consideration by arbitrazh courts of the Russian Federation of cases related to protection of intellectual property in 2012–2013) available from: http://www.arbitr.ru/

51 Federal Law No. 137-FZ of 27 July 2006 amending the Tax Code to include compulsory pre-judiciary administrative consideration of disputes related to tax offenses, the most numerous category of claims against non-normative decions of the state bodies.

52 On this point see Hendley (1999). In Russia, the scholarly discussion of ‘demand for law’ is focused more on procedural and less on sociological questions, and it addresses the need to fill legal gaps. This theory can be traced back to the Soviet legal scholarship. See recently Tumanov (2009). For general analysis see Solomon (2004). RCC Rulings on Merits No. 3-P of 25 April 1995; No. 11-P of 15 July 1999; No. 16-P of 13 December 2001; No. 12-P of 17 June 2004; No. 16-P of 11 November 2003; No. 21-P of 17 October 2012. On lack of legal certainty as a denial of equality before the law see Bondar (2011). See also Presnyakov (2009); Gadjiyev and Kovalenko (2012).

53 See for discussion Hendley (1999, p. 92)

54 Vladimir Putin, *Annual Address to the Federal Council of the Russian Federation*. *Kremlin.ru,* 3 April 2001, available from: http://kremlin.ru/events/president/transcripts/21216.

55 ‘Otkrytoye pismo Vladimira Putina k rossiyskim izbiratelyam’ (Open letter from Vladimir Putin to the Russian voters) *Kommersant* 32, 25 February 2000. Legal reform formed part of a wider capacity-building strategy to reinforce the national government, involving, for example, formation of *United Russia*, imposition of stricter control on regional elites, reduction of regional corruption.

56 See Explanatory note to the draft federal law No. 381232–4 (Code of Administrative Proceedings), available from: http://asozd2.duma.gov.ru/main.nsf/(Spravka)?OpenAgent&RN=381232-4

57 2007 Report of the Nationwide Mass Survey of Russian Citizens (WCIOM) *Otsenka deyatelnosti sudov v Rossiyskoy Federatsii* (Assessment of the activity of courts in the Russian Federation). Available from:
https://wciom.ru/database/open_projects/otsenka_deyatelnosti_sudov_v_rossii/

58 This trend is well-documented by Russian researchers and politicians of that time. See, for example, ‘Deputat Gosdumy V. Ilyukhin: Zakony yest, a zakonnosti net’ (Duma Deputee V. Ilyukhin: there are laws, but no rule of law) *Sovetskaya Rossiya* 32–33 (12658), 12 March 2005, available from: http://www.sovross.ru/old/2005/32/32_2_3.htm.

59 See discussion in Korchagin, Nomokonov and Shulga (1995).

60 Between 2007 and 2016, the number of cases whereby the applicants appeled to courts of general jurisdiction for debt recovery has increased by 700% (from just over 440,000 cases to well over 3 million cases per year). Recovered sums have increased in 17 times, even considering the financial crisis of 2008 (from 48 million roubles to 840 billion roubles per year). Source for statistical information: www.cdep.ru. Legislative changes included the creation of federal bailiffs service (Federal Law No. 118-FZ of 21 July 1997 ‘O sudebnykh pristavakh’ (On Bailiffs)) and establishing procedural framework for their operation (Federal Law No. 229-FZ of 2 October 2007 ‘Ob ispolnitelnom proizvodstve’ (On the implementation proceedings). The list of documents allowing bailiffs to ensure the execution of court decisions and extra-judicial decisions keeps expanding. For example, in 2008 the list was amended to include executive inscription of a notary public—an extra-judiciary mechanism to ensure the referral of debt to the bailiff system. Since 2016 private debt collectors have to be licensed (Federal Law No. 230-FZ of 3 July 2016 ‘O zashchite prav i zakonnykh interesov fizicheskikh lits pri osushchestvlenii deyatelnosti po vozvratu prosrochennoy zadolzhennosti i o vnesenii izmeneniy v Federalnyy zakon “O mikrofinansovoy deyatelnosti i mikrofinansovykh organizatsiyakh” (On the protection of the rights and legitimate interests of individuals in the implementation of activities for the repayment of debt and on amending the Federal Law “On microfinance activities and microfinance organizations”).

61 ‘V KGI predstavili doklad “Severnyy Kavkaz i sovremennaya model demokraticheskogo razvitiya” (The Committee of Civil Initiatives presented its report ‘North Caucasus and the contemporary model of democratic development’) *Novoye Delo*, 1 April 2016, available from: https://ndelo.ru/detail/v-kgi-predstavili-doklad-.

62 Although, in some regions such ‘community courts’ are still considered contradictory to Article 118 (1) of the Russian Constitution according the exclusivity of justice to state courts only, see St. Petersburg City Court Decision No. 2146 of 16 February 2011.

63 Reportedly, the Chechens started filing claims in courts for the first time in ten years when in early 2016 more than 80% of rural population was deprived of its gas supply by the republican authorities. Court claims were (successfully) used as a last resort after letters to the Chechen President Kadyrov did not bring the expected results.

64 ‘Sud s otkrytymi glazami. Krovnaya mest v Chechne’ (Court with open eyes: blood feud in Chechnya) *OnKavkaz*, 13 September 2013, available from: http://onkavkaz.com/articles/946-sud-s-otkrytymi-glazami-krovnaja-mest-v-chechne.html

65 ‘Sudebnaya vlast v Chechne sostoyalas i dostoyno spravlyayetsya so svoimi zadachami’ (The judiciary branch in Chechnya has finally formed and adequately copes with its tasks) *Grozny-Inform*, 31 January 2011, available from: http://www.grozny-inform.ru/news/express/23024/

66 Alexander Bogomolov, ‘Ukaz Prezidenta—bolshoy podarok. Vladimir Putin naznachil predsedatelya Verkhovnogo suda Chechni’ (The Presidential decree is a great gift: Vladimir Putin has appointed the Chairman of the Supreme Court of the Chechen Republic) *Izvestia*, 23 March 2003, available from: http://www.memo.ru/hr/hotpoints/N-Caucas/ch99/200303/030323iz.htm

67 The growth of regional autonomy in Russia became evident soon after Stalin’s death. See Bahry (1987).

68 Yeltsin’s support for the sovereignty of the republics began as early as 1989. See Tolz (1990)

69 In 1990, Yeltsin famously instructed subjects of the Republic to ‘take as much sovereignty as you can swallow.’ Later in the Treaty of Federation of 1992 he claimed authority to appoint regional governors. See Moraski (2006, pp. 15, 17).

70 Indicatively, some regions have even tried to introduce their own regional judicial systems, such as Bashkortostan and Ingushetia Republics. See Pavlikov (2004).

71 See Konitzer and Wergren (2006).

72 See discussion of relevant historical background in Brubaker (1994); Martin (2001); Motyl (2001); Roshwald (2001); Riga (2012).

73 See Willerton (1992); Walker (2003).

74 See on this point in general Arieli (1961, p. 35); Rokkan (1961, p. 138); Caramani (2004); Schattschneider (1988, pp. 89, 90). On weak political nationalization in Russia, see Golosov (2015).

75 See Reuter and Robertson (2012).

76 See Saikkonen (2017).

77 See Hale (2006); Moraski (2006); Goode (2011).

78 One of Putin’s most important early orders was Decree No. 1486 of 10 August 2000 ‘O dopolnitelnykh merakh po obespecheniyu yedinstva pravovogo prostranstva Rossiyskoy Federatsii’ (On Additional measures to ensure the unity of the legal space in the Russian Federation). See Sharlet (2001); Kahn (2002).

79 President of the Russian Federation Annual Address to the Federal Council of 8 July 2000, *Rossiyskaya Gazeta* 133, 11 July 2000.

80 Article 124 of the Russian Constitution, Article 1 of the Federal Law No. 30-FZ of 10 February 1999 ‘O finansirovanii sudov Rossiyskoy Federatsii’ (On the financing of courts of the Russian Federation). Justices of the peace receive salaries and benefits from the federal budget, however, the regional budgets provide for the material and technical support of the offices of justices of the peace (Article 10(3) of the Federal Law No. 188-FZ of 17 December 1998 ‘O mirovykh sudyakh v Rossiyskoy Federatsii’ (On justices of the peace in the Russian Federation).

81 Article 6(3) of the Federal Law No. 3132–1 of 26 June 1992 ‘O statuse sudey v Rossiyskoy Federatsii’ (On the status of judges in the Russian Federation).

82 Article 6 of the Federal Law No. 188-FZ of 17 December 1998 ‘O mirovykh sudyakh v Rossiyskoy Federatsii’ (On justices of the peace in the Russian Federation).

83 Report of the Chairman of the Higher Arbitrazh Court of the Russian Federation Veniamin Yakovlev at the meeting of chairmen of Russian arbitrazh courts, 16 February 1999, available from: http://www.arbitr.ru/press-centr/news/totals/report99/990216-1.htm

84 Art. 3(1) CACP.

85 Art. 2(1) APC.

86 Supreme Court Plenum Resolution No. 36 of 27 September 2016 ‘O nekotorykh voprosakh primeneniya sudami Kodeksa administrativnogo sudoproizvodstva Rossiyskoy Federatsii’ (On some issues of application by courts of the Code of Administrative Proceedings of the Russian Federation).

87 *Obzor sudebnoy praktiki Verkhovnogo Suda Rossiyskoy Federatsii* (Summary of judicial practice of the Supreme Court of the Russian Federation) No. 3 (2015) adopted by the Presidium of the Supreme Court on 25 November 2015, pp. 82–92.

88 See for example, ‘Voprosy sudebnoy praktiki primeneniya Kodeksa administrativnogo sudoproizvodstva Rossiyskoy Federatsii’ (Issues of judicial practice in the application of the Code of Administrative Proceedings of the Russian Federation), Sakhalin Oblast Court, available from: http://oblsud.sah.sudrf.ru/modules.php?name=docum_sud&id=223

89 Search engine of the unified database of Russian courts, available from: https://bsr.sudrf.ru/bigs/portal.html.

90 See, for example, *Kakova tsel strategicheskogo sudebnogo protsessa?* (Why should you consider bringing strategic litigation?) Child Rights International Network, available from: https://www.crin.org/ru/biblioteka/publikacii/kakova-cel-strategicheskogo-sudebnogo-processa; *Strategic Litigation Textbook* (ERRC/Interights/MPG, 2004).

91 Official website http://sutyajnik.ru/.

92 Ibid.

93 *Sutyazhnik v. Russia* (Judgment of 23 July 2009).

94 *Rakevitch v. Russia* (Judgment of 28 October 2003).

95 RCC Ruling on Merits No. 8-P of 22 April 2013.

96 In *Khoroshenko v. Russia* (Application no. 41418/04, Judgment of 30 June 2015) the Court found a violation of Article 8 rights (respect for his private and family life), as a result of the application of the strict regime in the special-regime penal colony in his case (§ 149). The Court concluded that ‘the interference with the applicant’s private and family life resulting from such a low frequency of authorised visits, solely on account of the gravity of a prisoner’s sentence was, as such, disproportionate to the aims invoked by the Government’ (§ 146).

97 RCC Ruling on Merits No. 24-P of 15 November 2016.

98 RCC Rulings on Admissibility No. 257-O of 24 May 2005 and No. 248-O of 9 June 2005.

99 Articles 125 and 127 of the Penitentiary Code of the Russian Federation No. 1-FZ of 8 January 1997.

100 Federal Law No. 292-FZ of 16 October 2017 'O vnesenii izmeneniy v Ugolovno-ispolnitelniy kodeks Rossiyskoy Federatsii’ (On Amendments to the Penal Enforcement Code of the Russian Federation).

101 See Burkov (2017).

102 See other examples of strategic litigation in Burkov (2010b).

103 Plenum of the Supreme Court Resolution No. 8 of 31 October 1995 ‘O nekotorykh voprosakh primeneniya sudami Konstitutsii Rossiyskoy Federatsii pri osushchestvlenii pravosudiya’ (On some issues of application by the courts of the Constitution of the Russian Federation in the administration of justice). Such usage of the Plenary Resolution was confirmed, for example, by a judge of Volzhskiy city court in Volgograd Oblast in 2000, interview available from: https://www.lawmix.ru/comm/6536.

104 See, among a number of other decisions, Supreme Court Decision No. 18-AD14–58 of 7 November 2014; Abinskiy District Court of Krasnodarsky Krai Decision No. 5–116/14 of 11 April 2014. On deportation of HIV-infected migrants, see RCC Ruling on Merits No. 4-P of 12 March 2015; RCC Ruling on Admissibility No. 155-O of 12 May 2006.

105 Supreme Court Plenum Ruling No. 5 of 24 March 2005 ‘O nekotorykh voprosakh, voznikayushchikh u sudov pri primenenii Kodeksa Rossiyskoy Federatsii ob administrativnykh pravonarusheniyakh’ (On some issues arising from application of the Code on Administrative Offenses by courts). (amended on 19 December 2013): Para. 23.1.

106 Nalogoviy kodeks Rossiyskoy Federatsii. Chast Pervaya (Tax Code of the Russian Federation. Part One) No. 146-FZ of 31 July 1998; Part Two, No. 117-FZ of 5 August 2000.

107 RCC Rulings on merits No. 19-P of 1 July 2015; No. 33-P of 25 December 2012.

108 RCC Ruling on merits No. 6-P of 31 March 2015.

109 RCC Ruling on merits No. 5-P of 17 March 2009.

110 ‘Sud v Voronezhe prekratil administrativnoye proizvodstvo za soglasovannyye mitingi’ (The court in Voronezh has stopped the administrative proceedings on a permitted public event). *OVD-info*, 8 February 2017, available from: https://ovdinfo.org/express-news/2017/02/08/sud-v-voronezhe-prekratil-administrativnoe-proizvodstvo-za-soglasovannye.

111 In Burkov (2010b), it is argued that sometimes judges need to be ‘trained’ to apply the Convention. On the other hand, most of them are well-trained and intentionally do not refer to the Convention in cases when such reference may be contrary to their own inclination regarding the potential outcome of the case (p. 176). Indicatively, in the legal practice of Burkov’s strategic litigation NGO there was not a single case when the judges initiated reference to the Convention themselves. Each time it was the work of the lawyers to convince them to apply a particular norm in the Convention (p. 178).

112 RCC Ruling on Merits No. 21-P of 14 July 2015 established the supremacy of the Constitution. This was later confirmed by Federal Constitutional Law No. 7-FKZ of 14 December 2015. In fact, even the RCC has used the ‘Supremacy’ ruling to strengthen the position of the Convention and not to undermine it. For example, in one of its own rulings adopted after 2015, the RCC stated: ‘The Convention and the ECtHR judgments, to the extent that they interpret the conventional rights and freedoms in line with universal principles and norms of international law, constitute an inseparable part of the Russian legal system. Therefore, federal legislator as well as law-applying bodies, should take into consideration the norms of the Convention in drafting and applying new legislation.’ (RCC Ruling on Admissibility No. 117-O of 28 January 2016).

113 See item 5 of the Supreme Court of the Russian Federation Plenary Ruling No. 2 of 10 February 2009 (void since 27 September 2016 when a new Plenary Ruling No. 36 has clarified issues of application of similar provisions of the Code of Administrative Proceedings); and RCC Ruling on Merits No. 19-P of 18 July 2012.

114 Federal Law No. 80-FZ of 7 May 2013 ‘O vnesenii izmeneniy v statyu 5.59 Kodeksa Rossiyskoy Federatsii ob administrativnykh pravonarusheniyakh i stati 1 i 2 Federalnogo zakona “O poryadke rassmotreniya obrashcheniy grazhdan Rossiyskoy Federatsii”’ (On amendments to article 5.59 of the Code of Administrative Offences and articles 1 and 2 of the Federal Law “On regulations concerning consideration of Russian Citizens’ petitions”).

115 The use of ECtHR precedents in resolving domestic cases is highly encouraged by the Supreme Court. For instance, 25% of the latest summary of judicial practice distributed by the Supremem Court among all Russian judges was dedicated to the jurisprudence of ECtHR, see *Obzor sudebnoy praktiki Verkhovnogo Suda Rossiyskoy Federatsii* (Summary of judicial practice of the Supreme Court of the Russian Federation) No. 4(16) approved by the Presidium of the Supreme Courn on 20 December 2016, pp. 141–190.

116 Supreme Court of the Russian Federation Plenum Ruling No. 21 of 27 June 2013 ‘O primenenii sudami obshchey yurisdiktsii Konventsii o zashchite prav cheloveka i osnovnykh svobod ot 4 noyabrya 1950 goda i Protokolov k ney’ (On the application by courts of general jurisdiction of the Convention for the Protection of Human Rights and Fundamental Freedoms of 4 November 1950 and the Protocols thereto).

117 RCC Ruling on Merits No. 15-P of 27 June 2012.

118 Federal Law No. 302-FZ of 30 December 2012.

119 See Eckstein (2004); Yershov (2006); Zorkin (2007).

120 See Pomeranz and Gutbrod (2012).

121 For the summary of the dispute, see Zorkin (2004); Aranovskiy and Knyazev (2013); Gadjiyev (2013).

122 See Ivanov (2010). The Higher Arbitrazh Court was abolished ‘for higher consistency of judicial practice’ by Law on Amendments to the Constitution No. 2-FKZ of 5 February 2014.

123 See Federal Arbitrazh Court of Far-Eastern Federal District, *Proyekt obzora sudebnoy praktiki po voprosam, svyazannym s rassmotreniyem del o priznanii nedeystvuyushchimi normativnykh pravovykh aktov* (Draft review of court practice on issues related to cases on invalidation of normative legal acts)*.* Available from: www.fasdvo.arbitr.ru/files/doc/proekt_po_npa.doc.

124 The RCC noted in Ruling on Admissibility No. 827-O of 28 May 2013, and, earlier in Ruling on Merits No. 30-P of 21 December 2011, that ‘recognition of prejudicial value of a court decision serves as a means of maintaining consistency of judicial practice and ensures the principle of legal certainty.’

125 This trend is most visible in the increase of criminal convictions for persons reposting allegedly ‘extremist’ content on social networks. Owing to the vague definition of extremist public expression, criminal proceedings are often initiated deliberately to silence opposition activists or opponents of Russia’s internal politics or territorial claims. Internet -related make up over 85% of all criminal convictions for extremism. In this context, the number of convictions increased from 626 in 2016 to 785 in 2017. In the first 6 months of 2018 over 300 people have been sentenced for reposts. For details see *Ofitsialnaya statistika pravoprimeneniya v sfere borby s ekstremizmom za 2017 god* (Official statistics on law enforcement in the field of combating extremism in 2017), SOVA, 27 April 2018, available from: https://www.sova-center.ru/racism xenophobia/news/counteraction/2018/04/d39283/ . For comment see *Srok za repost* (Imprisonment for a repost), Radio Svoboda, 9 January 2018, available from: https://www.svoboda.org/a/28943937.html.

126 See the account of ‘para-constitutionalism’ in Sakwa (2011, p. 47).

127 Colombian Constitutional Court SU-1150/00

128 In India, human rights litigation is encouraged so that, according to the Supreme Court, ‘the large masses of people’ can be integrated in the justice system. *S.P. Gupta* vs *President of India and ors*. on 30 December, 1981 1982 2 SCR 365 (Bhagwati J)

129 See *Griggs v Duke Power Co*, 401 US 424 (1971).

130 On weak systemic nationalization in the United States see Mickey (2015); Valelly (2004). On the structure-builiding force of rights litigation see Kaczorowski (1987); Graham (1990); Kelemen (2009).

131 On the United States in this regard see Skrentny (2002).

## References

[CR1] Abdrakhmanov R (2012). ‘Sverkhusileniye prezidentsko-pravitelstvennoy vlasti v rossiyskoy sisteme razdeleniya vlastey (2000–2010 gg.)’ (Over-strengthening of Presidential-Governmental power in the Russian system of separation of powers (2000–2010)). Kazanskiy Federalist.

[CR2] Aranovskiy K, Knyazev S (2013). ‘Sudba sudebnogo pretsedenta v romano-germanskom prave’ (The destiny of judicial precedent in Roman-Germanic law). Zhurnal konstitutsionnogo pravosudiya.

[CR3] Arieli, Y. (1964). *Individualism and Nationalism in American Ideology*. Cambridge: Harvard University Press.

[CR4] Artemova DI, Lichko EV (2014). ‘Vliyaniye sudebnoy politiki na sudebnoye reformirovaniye Rossii’ (The influence of judicial policy on the judicial reform in Russia). Vestnik Penzenskogo gosudarstvennogo universiteta.

[CR5] Artyushkina SV (2011). ‘Prichiny i usloviya samoupravnogo razresheniya imushchestvennykh i neimushchestvennykh sporov v Rossii (mery profilaktiki)’ (Reasons and conditions of self-judging resolution of property and non-monetary disputes in Russia (measures of prevention)). Pravovaya Kultura.

[CR6] Bahry D (1987). Outside Moscow. Power, politics, and budgetary policy in the soviet republics.

[CR7] Bakhrakh D (2008). ‘Etapy stanovleniya administrativnogo sudoproizvodstva v Rossii’ (Stages of evolution of administrative judicial proceedings in Russia). Rossiyskiy yuridicheskiy zhurnal.

[CR8] Bernard-Maugiron N, Bernard-Maugiron N (2008). The relationship between judges and human rights organizations during the 2005 elections and the referendum. Judges and political reform in Egypt.

[CR9] Bogaards M (2009). How to classify hybrid regimes? Defective democracy and electoral authoritarianism. Democratisation.

[CR10] Bondar, N. S. (2011). *Sudebnyy konstitutsionalizm v Rossii (Judicial constitutionalism in Russia)*. Moscow: Norma.

[CR11] Brown-Nagin T (2005). Elites, social movements and the law: the case of affirmative action. Columbia Law Review.

[CR12] Brubaker R (1994). Nationhood and the National Question in the Soviet Union and post-soviet Russia: an institutionalist account. Theory and Society.

[CR13] Burkov A (2010). Convention for protection of human rights in Russian courts.

[CR14] Burkov A (2010). Konventsiya o zashchite prav cheloveka v sudakh Rossii (convention for protection of human rights in Russian courts).

[CR15] Burkov, A. (2017) ‘V borbe za pravo obnyat muzha, otsa, syna’ (Fighting for the right to hug a husband, a father, a son). In T. Brower & A. Burkov (Eds.) *Kak polozhit chinovnika na lopatki, ili strategicheskiye sudebnye tyazhby: opyt raboty amerikanskih i rossiyskih sutyazhnikov po obschestvenno znachimym delam* (How to put an officer on the blades, or strategic litigation: the experience of American and Russian litigants on the public interest cases) (pp. 73–86). Moscow.

[CR16] Burstein P (1991). Legal mobilization as a social movement tactic: The struggle for equal employment opportunity. American Journal of Sociology.

[CR17] Caramani, D. (2004). *The Nationalization of Politics. The Formation of National Electorates*. Cambridge: Cambridge University Press.

[CR18] Chandler A (2014). Citizenship, social rights and judicial review in regime transition: the case of Russia. Democratization.

[CR19] Dahl R (1989). Democracy and its critics.

[CR20] Devlin J (1995). The rise of the Russian democrats. The causes and consequences of the elite revolution.

[CR21] Black DJ (1973). The mobilization of law. The Journal of Legal Studies.

[CR22] Easter G (1996). Personal networks and Postrevolutionary state building: Soviet Russia reexamined. World Politics.

[CR23] Easter GM (2008). The Russian state in the time of Putin. Post-Soviet Affairs.

[CR24] Eckstein, K. (2004). ‘Priznaniye obshchikh printsipov prava. Doktrina pravovogo gosudarstva’ (recognition of general principles of law. The doctrine of the rule of law state). In M.A. Mityukov, S.V. Kabyshev, V.K. Bobrova & A.V. Sycheva (Eds), *Obshchepriznannyye printsipy i normy mezhdunarodnogo prava, mezhdunarodnyye dogovory v praktike konstitutsionnogo pravosudiya. Materialy Vserossiyskogo soveshchaniya* (The universally recognized principles and norms of international law, international treaties in the practice of constitutional justice. Proceedings of the Russian nationwide symposium) (pp. 36–39). Moscow: Mezhdunarodnyye otnosheniya.

[CR25] El-Ghobashy M (2008). Constitutionalist contention in contemporary Egypt. American Behavioral Scientist.

[CR26] Epp CR (1998). The rights revolution. Lawyers, activists, and supreme courts in comparative perspective.

[CR27] Epp CR (2009). Making rights real. Activists, bureaucrats, and the creation of the legalistic state.

[CR28] Eskridge WN (2001). Channeling: identity-based social movements and public law. University of Pennsylvania Law Review.

[CR29] Feeley MM, Rubin EL (1998). Judicial policy making and the modern state. How the courts reformed America’s prisons.

[CR30] Francis MM (2014). Civil rights and the making of the modern American state.

[CR31] Gadjiyev G (2013). ‘Metodologicheskiye problemy “pretsedentnoy revolyutsii” v Rossii’ (Methodological problems of the “precedent revolution” in Russia). Zhurnal konstitutsionnogo pravosudiya.

[CR32] Gadjiyev GA, Kovalenko KA (2012). ‘Printsip pravovoy opredelennosti v konstitutsionnom pravosudii’ (The principle of legal certainty in constitutional justice). Zhurnal konstitutsionnogo pravosudiya.

[CR33] Garavito CR, Franco DR (2000). Cortes y cambio social – Cómo la Corte Constitucional transformó el desplazamiento forzado en Colombia.

[CR34] Gelman V (2015). Authoritarian Russia. Analyzing post-soviet regime changes.

[CR35] Giles MW, Lancaster TD (1989). Political transition, social development, and legal mobilization in Spain. The American Political Science Review.

[CR36] Gill G (2015). Building an authoritarian polity.

[CR37] Ginsburg T (2003). Judicial review in new democracies. Constitutional courts in Asian cases.

[CR38] Golosov, G. V. (2015). The idiosyncratic dynamics of party system nationalization in Russia. *Post-Soviet Affairs, 31*(5), 397–419.

[CR39] Goode JP (2011). The decline of regionalism in Putin’s Russia. Boundary issues.

[CR40] Gordeeva, D. M. (2013). ‘Administrativnoye sudoproizvodstvo v Rossii kak garantiya demokraticheskogo poryadka razresheniya sporov mezhdu grazhdanami i vlastyu’ (Administrative proceedings in Russia as a guarantee of democratic resolution of disputes between citizens and the authorities). In N.G. Pavlova, D.V. Chuhvichev, E.N. Trikoz & L.S. Nesterenko (Eds.), *Pravo—Obshchestvo—Gosudarstvo: Voprosy Teorii i Istorii: Sbornik Materialov Mezhvuzovskoy Studencheskoy Konferentsii* (pp. 14–17). Moscow: People’s Friendship University.

[CR41] Graham HD (1990). The civil rights era. Origins and development of National Policy 1960–1972.

[CR42] Granat N (1998). ‘Istochniki prava’ (Sources of law). Yurist.

[CR43] Greve MS (1989). The non-reformation of administrative law: standing to sue and public interest litigation in West German environmental law. Cornell International Law Journal.

[CR44] Grzymala-Busse A, Luong PJ (2002). Reconceptualizing the state: lessons from post-communism. Politics and Society.

[CR45] Hale HE (2006). Why not parties in Russia? Democracy, federalism and the state.

[CR46] Hale HE (2015). Patronal Politics. Eurasian regime dynamics in comparative perspective.

[CR47] Hanson P, Teague E (2005). Big business and the state in Russia. Europe-Asia Studies.

[CR48] Hassner P (2008). Russia’s transition to autocracy. Journal of Democracy.

[CR49] Hendley K (1999). Rewriting the rules of the game in Russia: The neglected issue of the demand for law. East European Constitutional Review.

[CR50] Hendley K (2002). Suing the state in Russia. Post-Soviet Affairs.

[CR51] Hendley K (2011). Varieties of legal dualism: making sense of the role of law in contemporary Russia. Wisconsin International Law Journal.

[CR52] Hendley K (2013). The puzzling non-consequences of societal distrust of courts: explaining the use of Russian courts. Cornell International Law Journal.

[CR53] Hualing F, Cullen R (2001). Climbing the *Weiquan* ladder: a radicalizing process for rights-protection lawyers. The China Quarterly.

[CR54] Hunt A (1990). Rights and social movements: counter-hegemonic strategies. Journal of Law and Society.

[CR55] Ivanov A (2010). ‘Rech o pretsedente’ (The speech on precedent). Pravo.

[CR56] Ivanov SM (2014). ‘K voprosu o vliyanii norm mezhdunarodnogo prava na sudebnuyu zashchitu v administrativnom sudoproizvodstve’ (On the impact of international law on judicial protection in administrative proceedings). Problemy prava.

[CR57] Kachanov RE (2008). ‘Sudebnyy normokontrol v sisteme mekhanizmov “strategicheskogo pravosudiya”’ (Judicial review in the mechanism of strategic litigation). Administrativnoye i munitstipalnoye pravo.

[CR58] Kaczorowski RJ (1987). To begin the nation anew: Congress, citizenship, and civil rights after the civil war. The American Historical Review.

[CR59] Korchagin, A. G., Nomokonov, V. A., & Shulga, V. I. (1995). *Organizovannaya prestupnost i borba s ney (Organised crime and countering it)*. Vladivostok: Far Eastern State University.

[CR60] Kahn J (2002). Federalism, democratization, and the rule of law in Russia.

[CR61] Kahn J, Trochev A, Balayan N (2009). The unification of law in the Russian Federation. Post-Soviet Affairs.

[CR62] Kapczynski A (2008). The access to knowledge mobilization and the new politics of intellectual property. The Yale Law Journal.

[CR63] Kelemen RD, Börzel TA, Cichowski RA (2003). The EU rights revolution: Adversarial legalism and European integration. The state of the European Union: Law, politics and society.

[CR64] Kelemen, D. R. (2009). *The Strength of Weak States: Adversarial Legalism in the US and the EU*. Available from: http://www.unc.edu/euce/eusa2009/papers/kelemen_10B.pdf. Last accessed on 11. 9. 2018

[CR65] Konitzer A, Wergren SK (2006). Federalism and political recentralization in the Russian Federation: United Russia as the Party of Power. Publius.

[CR66] Latorre Iglesias EL (2015). Litigio structural y experimentalismo jurídico. Análisis sociojurídico a los cambios generados por la sentencia T-025 en la población desplazada.

[CR67] Lauth, H.-J. (2015). The matrix of democracy: a three-dimensional approach to measuring the quality of democracy and regime transformations. Würzburger Arbeitspapiere zur Politikwissenschaft und Sozialforschung, Nr. 6.

[CR68] Lauth H-J, Sehring J (2009). Putting deficient *Rechtsstaat* on the research agenda: reflections on diminished subtypes. Comparative Sociology.

[CR69] Lebedev V (2000). ‘Ot idei sudebnogo normokontrolya k administrativnomu sudoproizvodstvu’ (From the idea of judicial review to administrative judicial proceedings). Rossiyskaya Yustitsia.

[CR70] Ledeneva A (2008). Telephone justice in Russia. Post-Soviet Affairs.

[CR71] Lutz EL, Sikkink K (2000). International human rights law and practice in Latin America. International Organization.

[CR72] Martin T (2001). The affirmative action empire. Nations and nationalism in the Soviet Union, 1923–1939.

[CR73] Mamykin, A.S. & Ryabtseva, Y.V. (eds) (2016). *Problemy optimizatsii sudebnoy yurisdiktsii i sudebnoy nagruzki na sudebnuyu sistemu v sovremennykh usloviyakh: materialy mezhdunarodnoy nauchno-prakticheskoy konferentsii: sbornik statey (Contemporary Problems of Optimization of Court Jurisdiction and Judicial Burden on the Judicial System: Proceedings of the International Scientific-Practical Conference, collection of articles)*. Moscow: RUJ.

[CR74] Mazmanyan A (2015). Judicialization of politics: the post-soviet way. International Journal of Constitutional Law.

[CR75] McCann MW (1986). Taking reform seriously. Perspectives on public interest litigation.

[CR76] McCann MW (1994). Rights at work. Pay equity reform and the politics of legal mobilization.

[CR77] McCann MW, Sarat A (2004). Law and social movements. Blackwell companion to law and society.

[CR78] McCann, M. W. (2008). Litigation and legal mobilization. In G. A. Caldeira, R. Daniel Kelemen, & K. E. Whittington (Eds.), *The Oxford handbook of law and politics* (pp. 522–540). Oxford: Oxford University Press.

[CR79] Merry SE (1990). Getting justice and getting even. Legal consciousness among working-class Americans.

[CR80] Merry SE (2005). Human rights and gender violence*:* Translating international law into local justice.

[CR81] Mickey R (2015). Paths out of Dixie*.* The democratization of authoritarian enclaves in America’s deep south*,* 1944-1972.

[CR82] Moraski B (2006). Elections by design. Parties and patronage in Russia’s regions.

[CR83] Motyl AJ (2001). Imperial ends. The decay, collapse, and revival or empires.

[CR84] Motyl AJ (2016). Putin’s Russia as a fascist political system. Communist and Post-Communist Studies.

[CR85] Moustafa T (2003). Law versus the state: the Judicialization of politics in Egypt. Law & Social Inquiry.

[CR86] Munck GL (2016). What is democracy? A reconceptualization of the quality of democracy. Democratization.

[CR87] Nettl JP (1967). Political mobilization: A sociological analysis of methods and concepts.

[CR88] O’Brien KJ, Li L (2004). Suing the local state: administrative litigation in rural China. The China Journal.

[CR89] Panunzio S (1933). Rivoluzione e costituzione (Problemi costituzionali della rivoluzione).

[CR90] Pavlikov S (2004). ‘Rossiyskiy federalizm: vliyaniye novykh aspektov na razvitiye sudebnoy sistemy’ (Russian federalism: impact of new aspects on the development of the judicial system). Rossiya i sovremennyy mir.

[CR91] Pedriana N, Stryker R (2004). The strength of a weak agency: enforcement of title VII of the 1964 civil rights act and the expansion of state capacity, 1965-1971. American Journal of Sociology.

[CR92] Petrone L (2011). Institutionalizing pluralism in Russia: a new authoritarianism?. Journal of Communist and Transition Politics.

[CR93] Petrov N, Lipman M, Hale HE (2014). Three dilemmas of hybrid regime governance: Russia from Putin to Putin. Post-Soviet Affairs.

[CR94] Pomeranz W, Gutbrod M (2012). The push for precedent in Russia’s judicial system. Review of Central and East European Law.

[CR95] Presnyakov MV (2009). ‘Pravovaya opredelennost kak sistemnoye kachestvo rossiyskogo zakonodatelstva’ (Legal certainty as a systemic quality of the Russian legislation). Zhurnal rossiyskogo prava.

[CR96] Reuter OJ (2010). The politics of dominant party: United Russia and Russia’s governors. Europe-Asia Studies.

[CR97] Reuter OJ, Robertson GB (2012). Subnational appointments in authoritarian regimes: evidence from Russian gubernatorial appointments. The Journal of Politics.

[CR98] Riga L (2012). The Bolsheviks and the Russian Empire.

[CR99] Ripoll JL, Sandvik K (2015). Shifting frames, vanishing resources, and dangerous political opportunities: legal mobilization among displaced women in Colombia. Law & Society Review.

[CR100] Risse T, Sikkink K, Risse T, Ropp SC, Sikkink K (1999). The socialization of international human rights norms into domestic practices: Introduction. The power of human rights: International norms and domestic change.

[CR101] Rivers J (2006). Proportionality and the variable intensity of review. The Cambridge Law Journal.

[CR102] Robertson GB (2011). The politics of protest in hybrid regimes. Managing dissent in post-communist Russia.

[CR103] Rosenberg, G. N. (1991). *The Hollow Hope. Can Courts Bring About Social Change?* Chicago: Chicago University Press.

[CR104] Rokkan, S. (1961). Mass Suffrage, Secret Voting and Political Participation. *European Journal of Sociology, 2*(01), 132–152.

[CR105] Roshwald A (2001). Ethnic nationalism and the fall of empires. Central Europe, Russia and the Middle East, 1914–1923.

[CR106] Rubin EL, Feeley MM (2002). Judicial policy making and litigation against the government. University of Pennsylvania Journal of Constitutional Law.

[CR107] Sabel CF, Simon WH (2004). Destabilization rights: how public law litigation succeeds. Harvard Law Review.

[CR108] Saikkonen IA-L (2017). Electoral mobilization and authoritarian elections: Evidence from post-soviet Russia. Government and Opposition.

[CR109] Sakwa R (2008). Putin and the oligarchs. New Political Economy.

[CR110] Sakwa R (2010). The dual state in Russia. Post-Soviet Affairs.

[CR111] Sakwa R (2011). The crisis of Russian democracy. The dual state, factionalism and the Medvedev succession.

[CR112] Schedler A, Schedler A (2006). The logic of electoral authoritarianism. Electoral authoritarianism: The dynamics of unfree competition.

[CR113] Scheingold SA (1974). The politics of rights. Lawyers, public policy and political change.

[CR114] Serkov P (2003). ‘Vvedeniye administrativnogo sudoproizvodstva—konstitutsionnyy dolg zakonodatelya’ (Introduction of administrative judicial proceedings is a constitutional duty of the legislators). Rossiyskaya yustitsiya.

[CR115] Sharlet R (2001). Putin and the politics of law in Russia. Post-Soviet Affairs.

[CR116] Schattschneider, E. E. (1988). *The Semisovereign People. A Realist’s View of Democracy in America, new edition*. Fort Worth: Harcourt Brace.

[CR117] Sheregi F (2002). Sotsiologiya prava. Prikladnyye issledovaniya (Sociology of Law. Applied Research).

[CR118] Shlapentokh, V. (1996). Early feudalism--the best parallel for contemporary Russia. *Europe-Asia Studies, 48*(3), 393–411 394, 396.

[CR119] Shmaliy OV (2013). ‘Kodeks administrativnogo sudoproizvodstva kak element konstitutsionnoy modeli pravosudiya’ (code of administrative proceedings as an element of the constitutional model of justice). Aktualnyye voprosy publichnogo prava. Nauchno-prakticheskiy zhurnal.

[CR120] Siegel, R. B. (2006). ‘Constitutional culture, social movement conflict and constitutional change: the case of the de facto era.’ California Law Review 94(5): 1323–1419.

[CR121] Sikkink K (1993). Human rights, principled issue-networks and sovereignty in Latin America. International Organization.

[CR122] Skoblikov PA (2000). ‘Samoupravstvo pri reshenii imushchestvennykh sporov: voprosy ugolovno-pravovogo protivodeystviya’ (Abuse of rights in the resolution of property disputes: questions of criminal legal counteraction). Izvestiya vysshikh uchebnykh zavedeniy. Pravovedeniye.

[CR123] Skoblikov, P. A. (2001). *Imushchestvennyye spory i kriminal v sovremennoy Rossii* (Property disputes and crime in contemporary Russia). Moscow: Delo.

[CR124] Skocpol T, Finegold K (1982). State capacity and economic intervention in the early new deal. Political Science Quarterly.

[CR125] Skrentny J (2002). The minority rights revolution.

[CR126] Smulovitz C, Sieder R, Schjolden L, Angell A (2005). Petitioning and creating rights: Judicialization in Argentina. The judicialization of politics in Latin America.

[CR127] Solomon PH (2004). Judicial power in Russia: through the prism of administrative justice. Law & Society Review.

[CR128] Solovieva, A. K. (2001). ‘Proizvodstvo po delam ob administrativnykh pravonarusheniyakh i administrativnoye sudoproizvodstvo: sootnosheniye ponyatiy’ (Administrative offenses proceedings and administrative judicial proceedings: correlation of terms). *Administrativnaya Otvetstvennost*, 56–59.

[CR129] Starilov, Y. (2003). *Ot administrativnoy yustitsii k administrativnomu sudoproizvodstvu (From administrative justice to administrative judicial proceedings)*. Voronezh: Voronezh State University.

[CR130] Starilov Y (2005). ‘Administrativnoye pravo kak sredstvo razrusheniya “sindroma bespraviya” v sovremennom pravovom gosudarstve’ (Administrative law as a means of destruction of “syndrome of lawlessness” in contemporary rule of law state). Zhurnal Rossiyskogo Prava.

[CR131] Starilov Y (2013). ‘Administrativnoye sudoproizvodstvo kak sposob povysheniya kachestva rossiyskogo gosudarstva’ (administrative proceedings as a way to improve the quality of the russian state). Aktualnyye voprosy publichnogo prava. Nauchno-prakticheskiy zhurnal.

[CR132] Starzhenetskiy V (2013). ‘Mezhdunarodnyye sudy i transformatsiya natsionalnykh pravovykh sistem’ (International courts and transformation of national legal systems). Mezhdunarodnoye pravosudiye.

[CR133] Sweet AS, Mathews J (2008). Proportionality balancing and constitutionalism. Columbia Journal of Transnational Law.

[CR134] Syukiyanen L (2014). ‘Sovmestim li shariat s sovremennym rossiyskim pravom?’ (Is Sharia compatible with contemporary Russian law?). Pravo. Zhurnal Vysshey Shkoly Ekonomiki.

[CR135] Tarrow S (1994). Power in movement. Social movements, collective action and politics.

[CR136] Taylor B (2011). State building in Putin’s Russia: policing and coercion after communism.

[CR137] Tikhomirov Y (2007). Law-based administration.

[CR138] Tikhomirova Y (2011). ‘Ponyatiye i priznaki proizvodstva po delam, voznikayushchim iz publichno-pravovykh otnosheniy kak vida grazhdanskogo sudoproizvodstva’ (The notion and characteristics of litigation arising from public law relations as a type of civil litigation). Vestnik RGGU. Seriya: Ekonomika. Upravleniye. Pravo.

[CR139] Tilly C (2000). Processes and mechanisms of democratization. Sociological Theory.

[CR140] Tilly C (2007). Democracy.

[CR141] Tolz V (1990). The USSR's emerging multiparty system.

[CR142] Tompson W (2002). Putin’s challenge: the politics of structural reform in Russia. Europe-Asia Studies.

[CR143] Trochev A (2012). Suing Russia at home. Problems of Post-Communism.

[CR144] Tsaliev A (2016). ‘Sudebnaya vlast kak obyazatelnyy atribut subyekta Rossiyskoy Federatsii; (Judicial power as a mandatory attribute of a subject of the Russian Federation). Zhurnal rossiyskogo prava.

[CR145] Tumanov DK (2009). ‘K voprosu o primenenii Konstitutsii Rossiyskoy Federatsii v sluchaye probelov v prave, a takzhe o roli Konstitutsionnogo Suda Rossiyskoy Federatsii v vyyavlenii takikh probelov’ (On the question of application of the Constitution of the Russian Federation in the event of gaps in the law, and the role of the Constitutional Court of the Russian Federation in identifying such gaps). Arbitrazhnyy i Grazhdanskiy Protsess.

[CR146] Valelly RM (2004). The two reconstructions. The struggle for black enfranchisement.

[CR147] Vanhala, L. (2011). *Making rights a reality? Disability rights activists and legal mobilization*. Cambridge University Press.

[CR148] Vanhala L (2012). Legal opportunity structures and the paradox of legal mobilization by the environmental movement in the UK. Law & Society Review.

[CR149] Walker EW (2003). Dissolution. Sovereignty and the breakup of the Soviet Union.

[CR150] White S (1990). Gorbachev in Power.

[CR151] Willerton J (1992). Patronage and politics in the USSR.

[CR152] Wilson BM, Cordero JCR (2006). Legal opportunity structures and social movements: The effects of institutional change on Costa Rican politics. Comparative Political Studies.

[CR153] Yakovlev A (2006). The evolution of business-state interaction in Russia: From state capture to business capture. Europe-Asia Studies.

[CR154] Yaroslavtsev SF, Yaroslavtseva SV (2012). ‘Informatsionno-pravovaya politika Rossii v sfere sudebnoy deyatelnosti: teoreticheskiye osnovy i voprosy realizatsii’ (Informational and legal policy of Russia in the sphere of judicial activity: theoretical foundations and issues of implementation). Yuridicheskaya nauka i pravookhranitelnaya praktika.

[CR155] Yeazell SC (2004). Brown, the civil rights movement and the silent litigation revolution. Vanderbilt Law Review.

[CR156] Yershov V (2006). Samostoyatelnost i nezavisimost sudebnoy vlasti Rossiyskoy Federatsii (Autonomy and independence of the judicial power of the Russian Federation).

[CR157] Zemans FK (1983). The neglected role of the law in the political system. The American Political Science Review.

[CR158] Zorkin V (2004). ‘Pretsedentniy kharakter resheniy Konstitutsionnogo Suda Rossiyskoy Federatsii’ (The precedent nature of the rulings of the Constitutional Court of the Russian Federation). Zhurnal rossiyskogo prava.

[CR159] Zorkin V (2007). *Rossiya i Konstitutsiya v XXI veke: vzglyad s Ilinki* (Russia and the Constitution in the XXI Century: View from Ilinka).

[CR160] Zverev DV, Kallistratova RF, Fokin MA (2006). ‘Novyye istochniki grazhdanskogo protsessualnogo prava v svete Yevropeyskoy konventsii o zashchite prav cheloveka i osnovnykh svobod’ (new sources of civil procedural law in light of the European Convention on Human Rights). Yevropeyskaya integratsiya i razvitiye tsivilisticheskogo protsessa v Rossii. Sbornik nauchnykh statey (The European integration process and the development of the civil law in Russia. Collection of articles).

